# The DNMT3A ADD domain is required for efficient *de novo* DNA methylation and maternal imprinting in mouse oocytes

**DOI:** 10.1371/journal.pgen.1010855

**Published:** 2023-08-01

**Authors:** Ryuji Uehara, Wan Kin Au Yeung, Keisuke Toriyama, Hiroaki Ohishi, Naoki Kubo, Hidehiro Toh, Isao Suetake, Kenjiro Shirane, Hiroyuki Sasaki

**Affiliations:** 1 Division of Epigenomics and Development, Medical Institute of Bioregulation, Kyushu University, Fukuoka, Japan; 2 Division of Gene Expression Dynamics, Medical Institute of Bioregulation, Kyushu University, Fukuoka, Japan; 3 Advanced Genomics Center, National Institute of Genetics, Mishima, Japan; 4 Department of Nutrition Science, Nakamura Gakuen University, Fukuoka, Japan; 5 Department of Genome Biology, Graduate School of Medicine, Osaka University, Suita, Japan; The University of Edinburgh MRC Human Genetics Unit, UNITED KINGDOM

## Abstract

Establishment of a proper DNA methylation landscape in mammalian oocytes is important for maternal imprinting and embryonic development. *De novo* DNA methylation in oocytes is mediated by the DNA methyltransferase DNMT3A, which has an ATRX-DNMT3-DNMT3L (ADD) domain that interacts with histone H3 tail unmethylated at lysine-4 (H3K4me0). The domain normally blocks the methyltransferase domain via intramolecular interaction and binding to histone H3K4me0 releases the autoinhibition. However, H3K4me0 is widespread in chromatin and the role of the ADD-histone interaction has not been studied *in vivo*. We herein show that amino-acid substitutions in the ADD domain of mouse DNMT3A cause dwarfism. Oocytes derived from homozygous females show mosaic loss of CG methylation and almost complete loss of non-CG methylation. Embryos derived from such oocytes die in mid-to-late gestation, with stochastic and often all-or-none-type CG-methylation loss at imprinting control regions and misexpression of the linked genes. The stochastic loss is a two-step process, with loss occurring in cleavage-stage embryos and regaining occurring after implantation. These results highlight an important role for the ADD domain in efficient, and likely processive, *de novo* CG methylation and pose a model for stochastic inheritance of epigenetic perturbations in germ cells to the next generation.

## Author summary

Epigenetic modifications of histones and DNA play important roles in gene regulation and embryonic development. We herein examine the role of histone-tail recognition by DNMT3A, the major DNA methyltransferase expressed in mammalian oocytes, in mediating *de novo* DNA methylation in this cell-type. The ADD domain is known to interact with histone H3 tail unmethylated at lysine 4. We found that amino-acid substitutions in the DNMT3A ADD domain disrupting the histone-tail recognition cause mosaic loss of DNA methylation in mouse oocytes, suggesting that this domain plays an important role in efficient, and likely processive, DNA methylation. Furthermore, the mosaic loss of DNA methylation in oocytes resulted in lethality of derived embryos during mid-to-late gestation, with stochastic multi-locus imprinting defects. The ADD mutant mice pose a model to study stochastic inheritance of epigenetic perturbations in germ cells to the next generation.

## Introduction

Establishment of the normal DNA methylation landscape in mammalian oocytes is crucial for maternal genomic imprinting and embryonic development [1–4]. The *de novo* methylation process involves recognition of histone modification state by the DNA methyltransferase DNMT3A and its cofactor DNMT3L. The Pro-Trp-Trp-Pro (PWWP) domain of DNMT3A interacts with histone H3 di-/tri-methylated at lysine-36 (H3K36me2/3) and limits ectopic methylation in both somatic cells and oocytes [5–11]. The ATRX-DNMT3-DNMT3L (ADD) domain, shared by DNMT3A and DNMT3L, interacts with histone H3 tail unmethylated at lysine-4 (H3K4me0) [12–16] and, consistent with this, a histone H3K4 demethylase KDM1B is required to establish DNA methylation at least at some imprinting control regions (ICRs) in mouse oocytes [17,18]. These findings suggest that recognition of histone H3K4me0 by the DNMT3A/DNMT3L complex is important for *de novo* DNA methylation in oocytes. Since mutations of the DNMT3A ADD domain are found in patients with Tatton-Brown-Rahman syndrome and hematologic malignancy [19–22], the ADD-H3K4me0 interaction is important not only in oocytes but also in somatic cells.

A previous study reported that the DNMT3A ADD domain normally blocks its own methyltransferase domain and, upon H3K4me0 binding, releases the autoinhibition *in vitro* [16]. In contrast, the domain does not interact with H3K4me2/3, excluding chromatin regions with these marks, such as active enhancers and promoters, from targets of DNA methylation [12–16]. Two aspartic acids (D529 and D531) in the ADD domain of human DNMT3A are important for both autoinhibition and H3K4me0 binding [16]. However, the exact role of the ADD-H3K4me0 interaction is not understood *in vivo*.

We herein report that substitutions of the aspartic acids in the mouse DNMT3A ADD domain cause dwarfism and female infertility. We further demonstrate that the DNMT3A ADD domain plays an important role in efficient, and maybe processive, *de novo* methylation and maternal imprinting in mouse oocytes. The stochastic loss of imprinting in derived embryos poses a model to study how partial changes in epigenetic modifications in germ cells can be inherited or eliminated during development.

## Results

### Generation and phenotype of *Dnmt3a*^*ADA*^ mice

**Fig 1:**
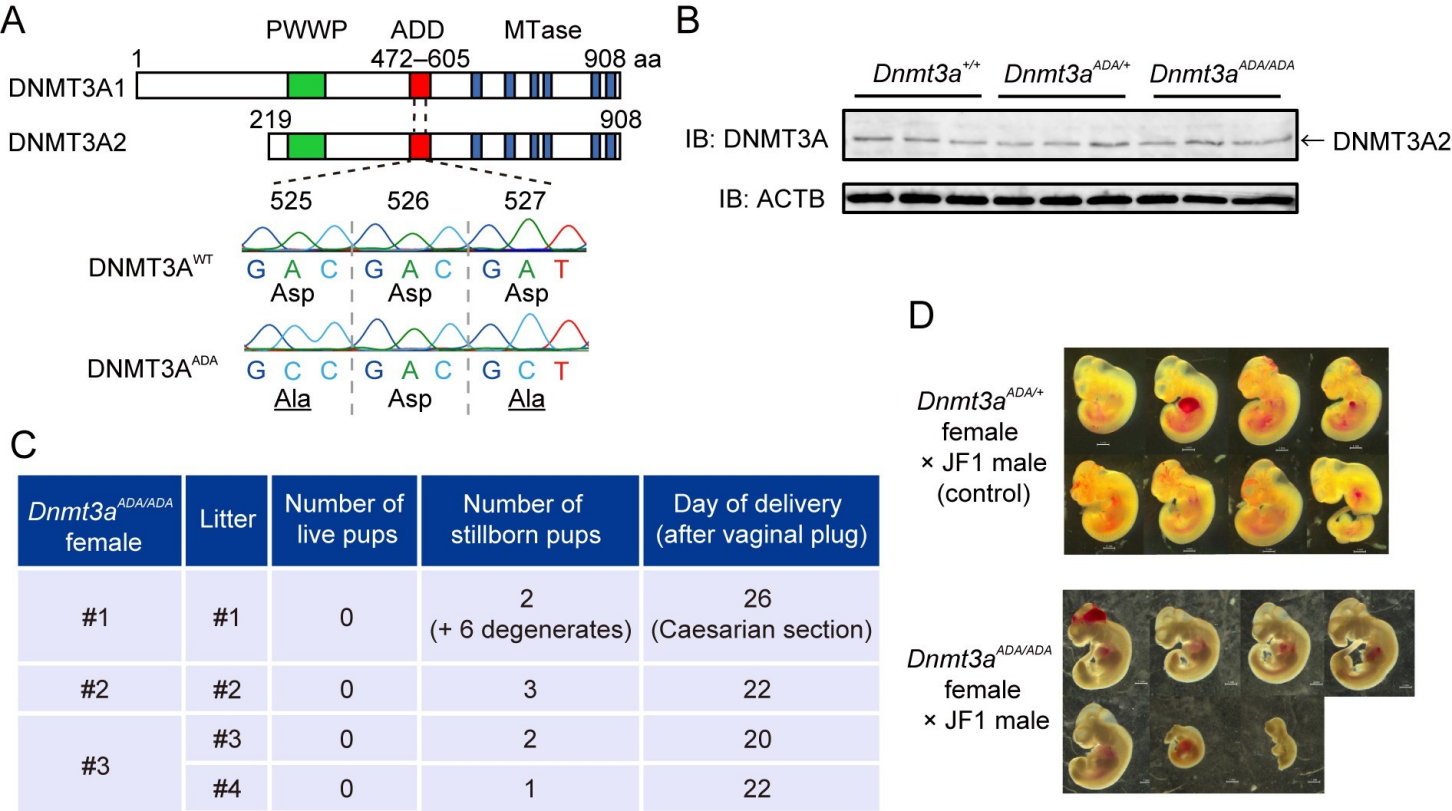
Generation and phenotype of *Dnmt3a*^*ADA*^ mice. (*A*) Structure of mouse DNMT3A isoforms and positions of D525A and D527A substitutions. Known domains (PWWP and ADD) and motifs of the methyltransferase (MTase) domain are indicated by colored boxes. A major isoform expressed in oocytes is DNMT3A2. An example of genotyping by Sanger sequencing is shown. (*B*) Western blotting of DNMT3A2 in FGOs. Proteins prepared from 50 FGOs were loaded for each genotype. ACTB is a loading control. (*C*) Fertility of *Dnmt3a*^*ADA/ADA*^ females crossed with a C57BL/6J male. Female #1 was subjected to Caesarian section, and two stillborn pups and six degenerated fetuses were obtained. (*D*) Representative images of embryos recovered at E10.5. Scale bar, 1 mm.

Two aspartic acids (D529 and D531) in the ADD domain of human DNMT3A are respectively important for both autoinhibition and H3K4me0 binding [16]. We generated mice carrying aspartic-acid-to-alanine substitutions at D525 and D527 (corresponding to human D529 and D531) using CRISPR/Cas9-mediated homology directed repair (see Materials and methods). We assumed that such DNMT3A would always adopt an active form but show reduced binding to H3K4me0 [16], the latter of which was confirmed by *in vitro* binding assay using wild-type and mutated recombinant ADD domains (S1A, and S1B Fig). The mutated allele and protein are called *Dnmt3a*^*ADA*^ and DNMT3A^ADA^, respectively (Fig 1A). We detected mutated DNMT3A2 protein in fully grown oocytes (FGOs), a major isoform expressed in this cell type, at a level comparable to wild-type protein (Fig 1B). *Dnmt3a*^*ADA/ADA*^ homozygous mice were obtained at a near Mendelian ratio and showed dwarf phenotype (S1C, S1D and S1E Fig), which contrasts with the overgrowth phenotype of Tatton-Brown-Rahman syndrome patients with mutations in this domain [22].

While homozygous males were fertile (litter size 7.1 ± 1.5 [n = 9 litters], versus 6.6 ± 1.4 for heterozygous control males [n = 9 litters]), homozygous females showed a low delivery rate per vaginal plug (4/7, versus 7/8 for heterozygous control females). Furthermore, the females suffered from prolonged pregnancy and gave no live pups with only a few stillborn pups (from 3 deliveries and one Caesarian section) (Fig 1C). To examine whether the phenotype is due to the mothers or the fetuses themselves, we performed *in vitro* fertilization (IVF) of *Dnmt3a*^*ADA/ADA*^ oocytes with wild-type sperm and transferred two-cell embryos to the oviducts of pseudo-pregnant females. The result showed a significant loss of embryos even in healthy mothers (S1 Table). When a mixture of embryos from wild-type and homozygous oocytes was transferred, all derived pups were from wild-type oocytes. Importantly, two live *Dnmt3a*^*ADA/ADA*^ oocyte-derived pups were obtained from a transferred female (S1 Table), suggesting that homozygous oocytes can occasionally support full development and that homozygous females do have delivery problems.

We then examined the timing of embryonic loss by crossing homozygous females with wild-type JF1 males [23]. While most embryos looked healthy at embryonic day 8.5–9.0 (E8.5–9.0), a small proportion was arrested at E10.5 (Fig 1D and S2 Table). The actual sizes of the E10.5 embryos from homozygous females were significantly smaller than those from wild-type or heterozygous females (S1F Fig). At E14.5 and E18.5, we observed frequent fetal resorption, and only about half of the implanted embryos developed normally to these stages (S2 Table). These results show that many embryos die during mid-to-late gestation in homozygous females.

### Impact of *Dnmt3a*^*ADA*^ mutation on CG and non-CG methylation in FGOs

**Fig 2:**
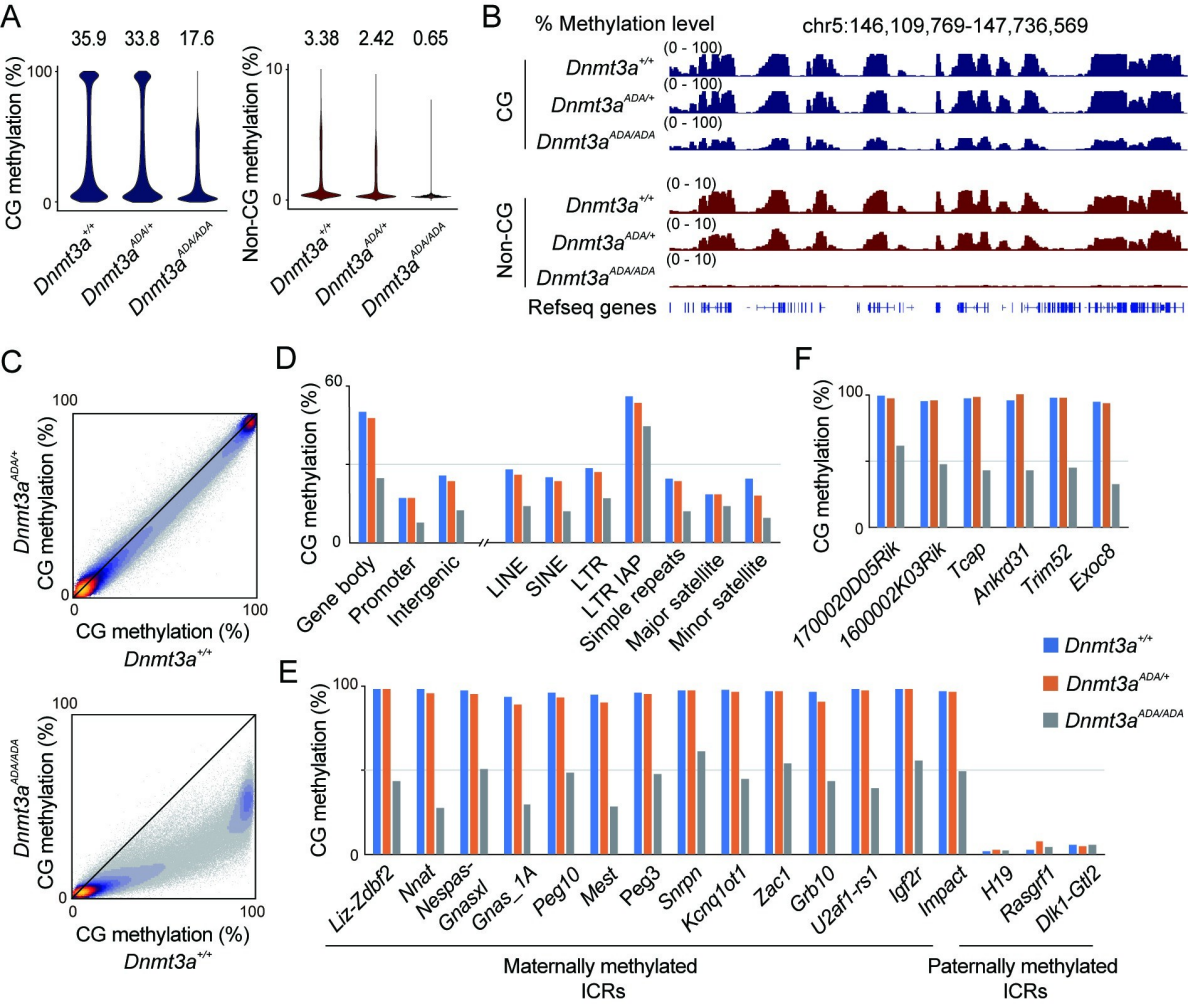
Impact of *Dnmt3a*^*ADA*^ mutation on CG and non-CG methylation in FGOs. (*A*) Violin plots showing distributions of CG and non-CG methylation levels of 10-kb genomic bins in pooled FGOs of indicated genotypes. The number above each plot indicates the global methylation level. Data obtained from biological replicates (each comprising 100 to 250 FGOs) were combined after confirmation of reproducibility (S3 Table). (*B*) Genome browser view of CG and non-CG methylation levels of 10-kb bins in FGOs. (*C*) Scatter plots comparing the CG methylation levels of 10-kb bins between FGOs of indicated genotypes. (*D*) CG methylation levels of indicated genomic annotations including various repeats in FGOs. Gene bodies and intergenic regions include repeats. (*E*) CG methylation levels of maternally and paternally methylated ICRs in FGOs of indicated genotypes. (*F*) CG methylation levels of promoter CpG islands that are normally methylated in FGOs. Six representative islands among those that we identified to be methylated in wild-type FGOs (n = 251) are shown.

We next examined the DNA methylation landscape of oocytes by whole-genome bisulfite sequencing (WGBS) with pooled FGOs (S3 Table). Homozygous FGOs showed nearly 50% reduction in global CG methylation compared to wild-type and heterozygous FGOs and almost complete loss of non-CG methylation (Fig 2A) (non-CG methylation of homozygous FGOs was close to the bisulfite conversion error rate; S3 Table). The methylation loss in homozygous FGOs occurred across the genome (Fig 2B and 2C). Among the different genomic annotations, intracisternal A particle elements, a class of endogenous retrovirus, were the least affected (Fig 2D), perhaps consistent with their preferential methylation and resistance to demethylation [24–29]. Notably, maternally methylated ICRs and promoter CpG islands that are fully CG methylated in wild-type FGOs showed 30%-60% reduction in CG methylation (Fig 2E and 2F). The examination of individual WGBS reads mapping to the ICRs revealed partial and mosaic CG methylation loss in each DNA template (S2 Fig). These results suggest that the establishment of the normal CG and non-CG methylation landscape is impaired in homozygous FGOs.

### Efficient, and likely processive, CG methylation is compromised in *Dnmt3a*^*ADA*^ mutant FGOs

**Fig 3:**
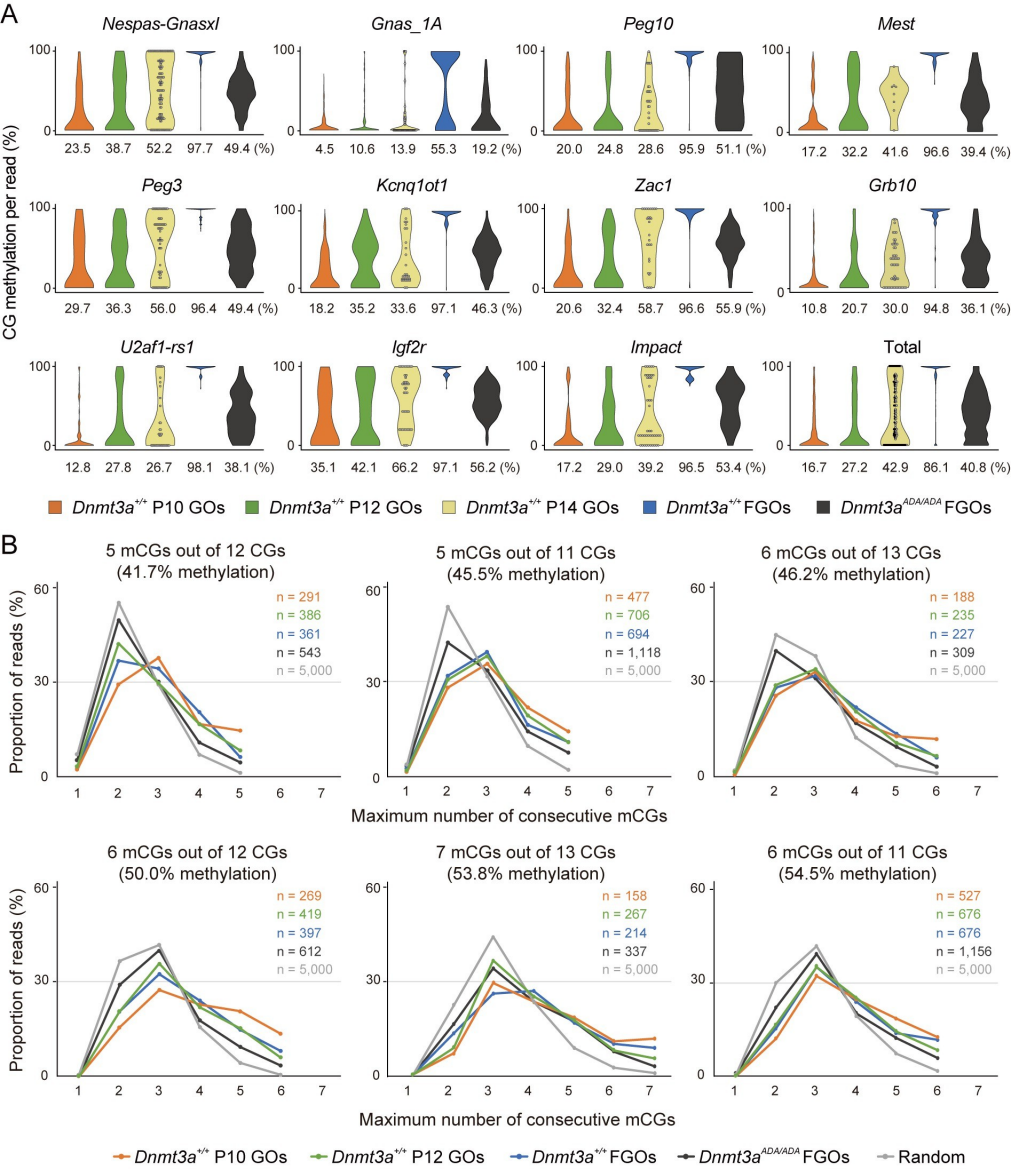
The ADD-H3K4me0 interaction supports efficient, and likely processive, DNA methylation. (*A*) Violin plots showing the distributions of CG methylation levels of individual WGBS reads (~100 nucleotides in length) at each maternally methylated ICR in wild-type GOs and wild-type and homozygous FGOs. Data obtained from biological replicates (each comprising 150 to 250 P12 GOs and 100 to 250 FGOs) were combined after confirmation of reproducibility (S3 Table). Published data were used for wild-type GOs collected at P10 [30] and P14 [31] (diameter 60–70 μm). The global methylation level of wild-type GOs at P10, P12, and P14 is 9.6%, 12.8%, and 22.9%, respectively. The global methylation level of wild-type and homozygous FGOs is 35.9% and 17.6%, respectively. Reads containing more than 4 CG sites were used. (*B*) Comparisons of the longest stretches of methylated CG in individual WGBS reads in wild-type GOs and wild-type and homozygous FGOs. Reads with the same number of CGs and methylated CGs (thus the same CG methylation ratio) were extracted from the WGBS reads. Proportions of WGBS reads with the indicated largest number of consecutive methylated CGs detected in each extracted read group are shown. Gray lines show the distribution of longest stretch lengths expected by mutually independent methylation of CG sites with the same methylation ratio. The WGBS data from P14 GOs was not used due to the small data size. See above for the global methylation level of each sample. mCGs, methylated CGs.

The mosaic loss of CG methylation in *Dnmt3a*^*ADA/ADA*^ FGOs suggests that the ADD domain may play an important role in efficient, and possibly processive, methylation in oocytes. To gain insights into how this domain controls *de novo* methylation, we compared the distribution of CG methylation levels of individual WGBS reads at the maternally methylated ICRs in homozygous FGOs with that of wild-type growing oocytes (GOs), in which *de novo* methylation is still ongoing [4]. As expected, we observed progressive CG methylation of the ICRs in GOs obtained at postnatal day 10 (P10) [30], P12, and P14 (diameter 60–70 μm) [31] (Fig 3A). Interestingly, the methylation levels of the individual WGBS reads often showed bimodal distribution at later stages (especially at P14; Fig 3A), suggesting that *de novo* ICR methylation tend to occur at multiple, proximal CG sites (processive methylation). By contrast, homozygous FGOs, which had CG methylation levels not very different from P12 and P14 GOs, showed a simple distribution suggestive of random methylation (Fig 3A). These results suggest that DNMT3A^ADA^ likely have a problem in processive CG methylation.

To investigate this possibility further, we selected WGBS reads containing exactly the same number of CGs and the same number of methylated CGs (thus the same CG methylation ratio) from the entire genome of wild-type GOs at P10 and P12, and wild-type and homozygous FGOs. We then determined the longest stretches of methylated CGs (or the largest number of consecutively methylated CGs) in individual reads. This analysis showed that, in each WGBS read group with the same CG number and the same CG methylation ratio, the methylated CG stretches found in wild-type GOs and FGOs were clearly longer than those expected by mutually independent methylation of CG sites with the same methylation ratio (Fig 3B and S4 Table). This suggests the occurrence of processive CG methylation in wild-type oocytes. In contrast, homozygous FGOs had a stretch length distribution closest to that of independent methylation (Fig 3B), which is supported by the Kullback-Leibler divergences (S5 Table), suggesting that methylation mediated by DNMT3A^ADA^ is more like distributive than processive *in vivo*. Although our analysis was limited by the number of CGs contained in individual reads generated by short-read WGBS, these results further support the idea that processive CG methylation is compromised in homozygous FGOs.

### Stochastic CG methylation loss at maternally methylated ICRs in derived embryos

**Fig 4:**
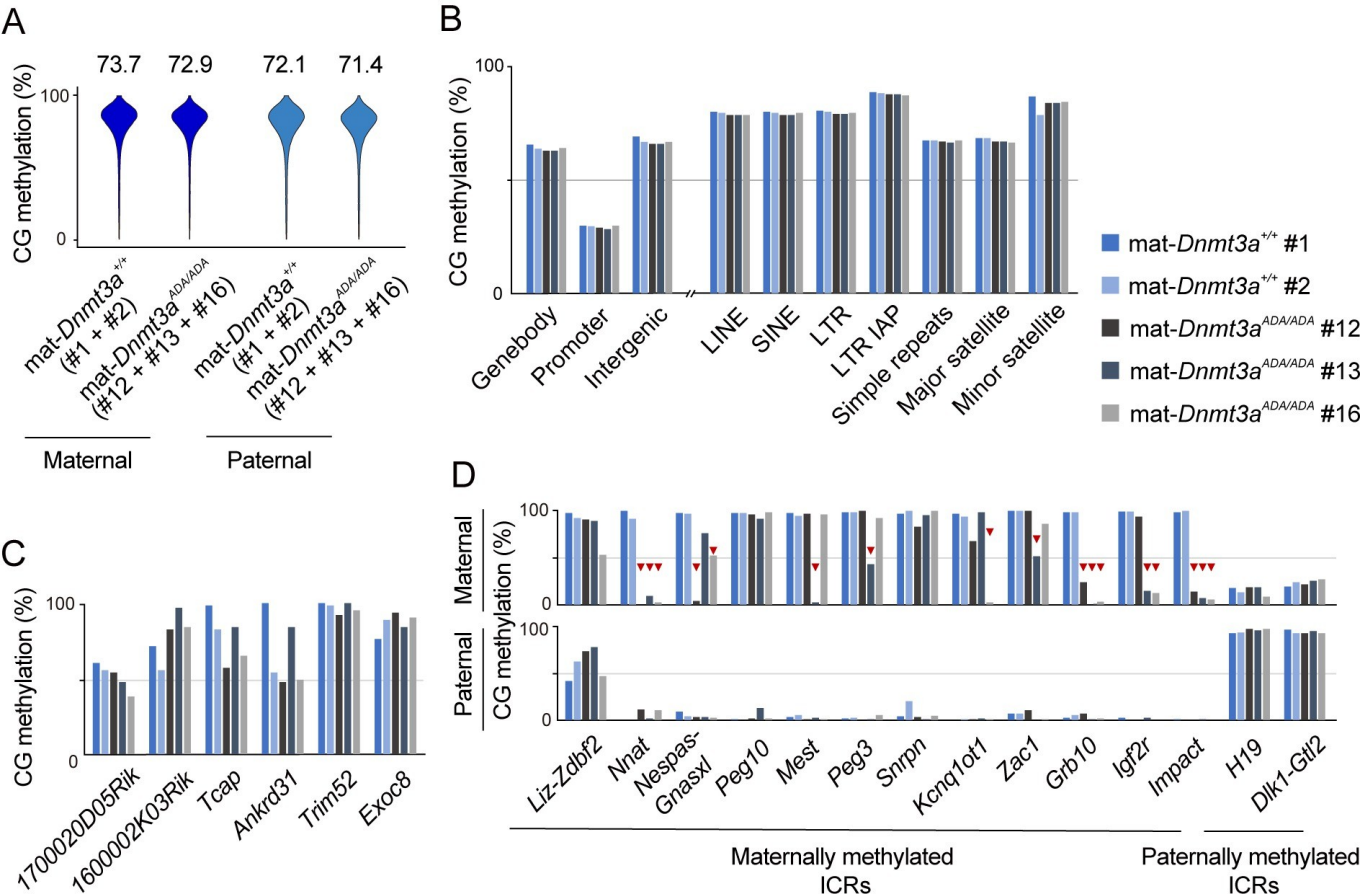
Stochastic CG methylation loss at maternally methylated ICRs in embryos. (*A*) Violin plots showing the distributions of CG methylation levels of 10-kb genomic bins in the maternal and paternal genomes of E10.5 embryos. The maternal genotype and embryo ID are indicated. Only SNP-containing reads were used for this analysis. Data from embryos of the same genotype were combined. The number above each plot indicates the global CG methylation level. (*B*) CG methylation levels of indicated genomic annotations including various repeats in individual embryos. (*C*) CG methylation levels of promoter CpG islands that are normally methylated in FGOs in individual embryos. The islands are the same ones as in Fig 2F (*D*) CG methylation levels at the maternally and paternally methylated ICRs in the maternal and paternal genomes of individual embryos. ICRs with more than 10 cumulative CG counts (redundant) in SNP-containing reads were analyzed. Red triangles indicate stochastic CG methylation loss.

To examine the inheritance of decreased CG methylation, we examined three E10.5 embryos from *Dnmt3a*^*ADA/ADA*^ females (mat-*Dnmt3a*^*ADA/ADA*^ embryos #12, #13, and #16 in S3 Fig) and two embryos from wild-type females as controls (mat-*Dnmt3a*^+*/*+^ embryo #1 and #2). Single nucleotide polymorphisms (SNPs) between laboratory mice (C57BL/6J) and JF1 mice were used for allele-specific analyses [23]. The overall CG methylation level was similar in all embryos regardless of the genotype (S4 Fig and S3 Table), and a SNP-based analysis confirmed this for both parental genomes (Fig 4A and S3 Table). Various genomic annotations and specific CpG islands that showed reduced methylation in homozygous FGOs (Fig 2D and 2F) had normal methylation levels (Fig 4B and 4C), suggesting that the maternal genome regains the normal CG methylation level before this stage. We then examined the maternally methylated ICRs as their methylation loss could persist in embryos [1–3]. Twelve maternally methylated and two paternally methylated ICRs had informative SNPs, and strikingly, the maternal allele of the maternally methylated ICRs showed “stochastic” loss of CG methylation in embryos derived from homozygous females (Fig 4D). There was embryo-to-embryo variation as to which ICR loses methylation, and in each embryo, some maternally methylated ICRs lost methylation while others regained full methylation. Partial methylation loss was also observed (see *Nespas-Gnasxl*, *Peg3* and *Zac1* in Fig 4D). No change was observed in the paternally methylated ICRs.

### The altered expression of imprinted genes is correlated with loss of ICR methylation

**Fig 5:**
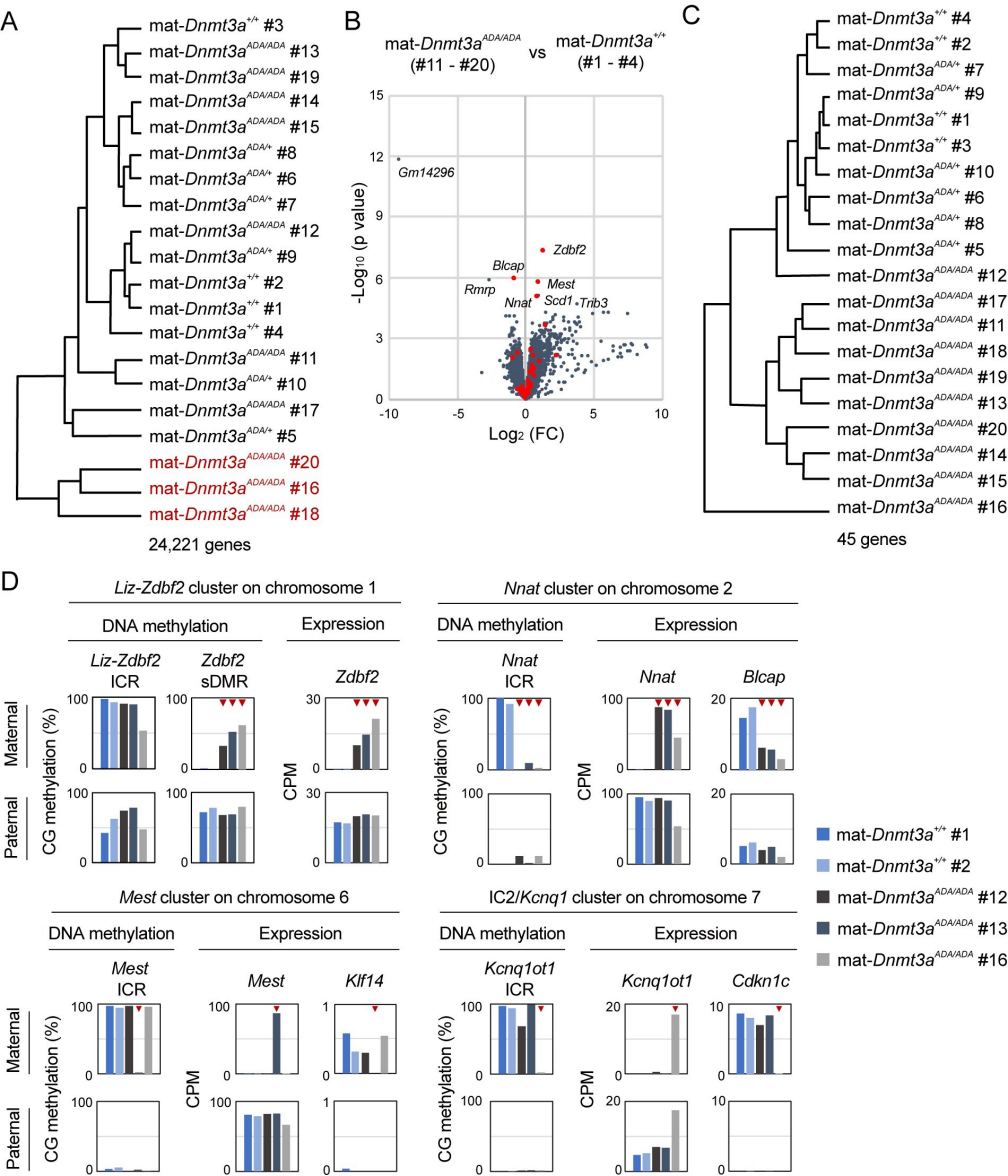
The altered expression of imprinted genes is correlated with loss of ICR methylation. (*A*) A cluster analysis of the transcriptomes from E10.5 embryos. The maternal genotype of the mother and embryo ID (#1–20) are indicated. The most retarded embryos in S3 Fig are indicated in red. (*B*) Volcano plot showing gene expression changes in embryos of indicated maternal genotypes. Red dots show the 45 maternally imprinted genes (S8 Table). (*C*) A cluster analysis using the expression profiles of the maternally imprinted genes. (*D*) Allelic CG methylation states of maternally methylated ICRs and allelic expression states of linked genes in representative imprinted gene clusters. SNP-based allele-specific analyses were conducted. Red triangles show stochastic CG methylation loss and the altered expression of the linked genes. sDMR, secondarily differentially methylated region. CPM, count per million.

The transcriptome was then examined by RNA sequencing (RNA-seq) in E10.5 mat-*Dnmt3a*^+/+^ (#1–4), mat-*Dnmt3a*^*ADA/*+^ (#5–10) and mat-*Dnmt3a*^*ADA/ADA*^ embryos (#11–20) (S6 Table). A cluster analysis of the transcriptomes of the individual embryos revealed that the similarity of the transcriptomes depends on the severity of defect (see S3 Fig for morphology) rather than the genotype (Fig 5A). However, when we attempted to identify genes differentially expressed between mat-*Dnmt3a*^+/+^ and mat-*Dnmt3a*^*ADA/ADA*^ embryos, eight genes were either upregulated or downregulated (FDR < 0.05), of which four were linked to the maternally methylated ICRs (*Zdbf2*, *Blcap*, *Mest*, and *Nnat*; maternally imprinted genes) (Fig 5B and S7 Table).

We therefore examined the expression of a total of 45 maternally imprinted genes in individual embryos: while their expression level was relatively constant in mat-*Dnmt3a*^+/+^ embryos, it varied greatly in mat-*Dnmt3a*^*ADA/ADA*^ embryos (S5A Fig and S8 Table). The imprinted genes showed either upregulation or downregulation, depending on their functional relationship with the linked ICR [32], in some of the mat-*Dnmt3a*^*ADA/ADA*^ embryos but not in others. A cluster analysis using the expression levels of the 45 imprinted genes clearly distinguished mat-*Dnmt3a*^*ADA/ADA*^ embryos from the other genotypes (Fig 5C). However, the exact number of the affected imprinted genes and degrees of their changes were different from embryo to embryo (S5B Fig). These results suggest that stochastic and varied misregulation of the maternally imprinted genes is a hallmark of embryos derived from homozygous females. *Gm14296* was a strongly downregulated non-imprinted gene (Fig 5B and S7 Table), and it turned out that this gene was secondarily regulated by imprinting through *Klf14*, a maternally imprinted gene linked to the *Mest* ICR [33] (Fig 5D), further corroborating the imprinting-related transcriptomic changes. Lastly, three mat-*Dnmt3a*^*ADA/ADA*^ embryos showing largest changes in the imprinted gene expression (#16, #18, and #20) (S5B Fig) were clearly underdeveloped (S3 Fig), suggesting a close link between the imprinted gene misregulation and phenotype.

We then identified allelic SNPs between the JF1 and C57BL/6J genomes in 16 maternally and 2 paternally imprinted transcripts (genes) and used them to examine whether or not the stochastic methylation loss was linked to the stochastic misregulation of the maternally imprinted genes *in cis*. It was found that, in each embryo, there was indeed a perfect link between the loss of methylation at the ICRs and misregulation of the linked genes exclusively in the maternal genome (Fig 5D and S5C Fig). The aberrant upregulation of *Zdbf2* was correlated with a gain of methylation at the secondarily differentially methylated region, not methylation loss at the ICR, consistent with the reported unique developmental regulation at this locus [34,35]. Since imprinted genes are crucial for embryonic development [36], the stochastic loss of maternal imprinting (and hence the difference in affected gene set) likely explains the loss of embryos at various stages.

### Stochastic loss of ICR methylation occurs during early development

**Fig 6:**
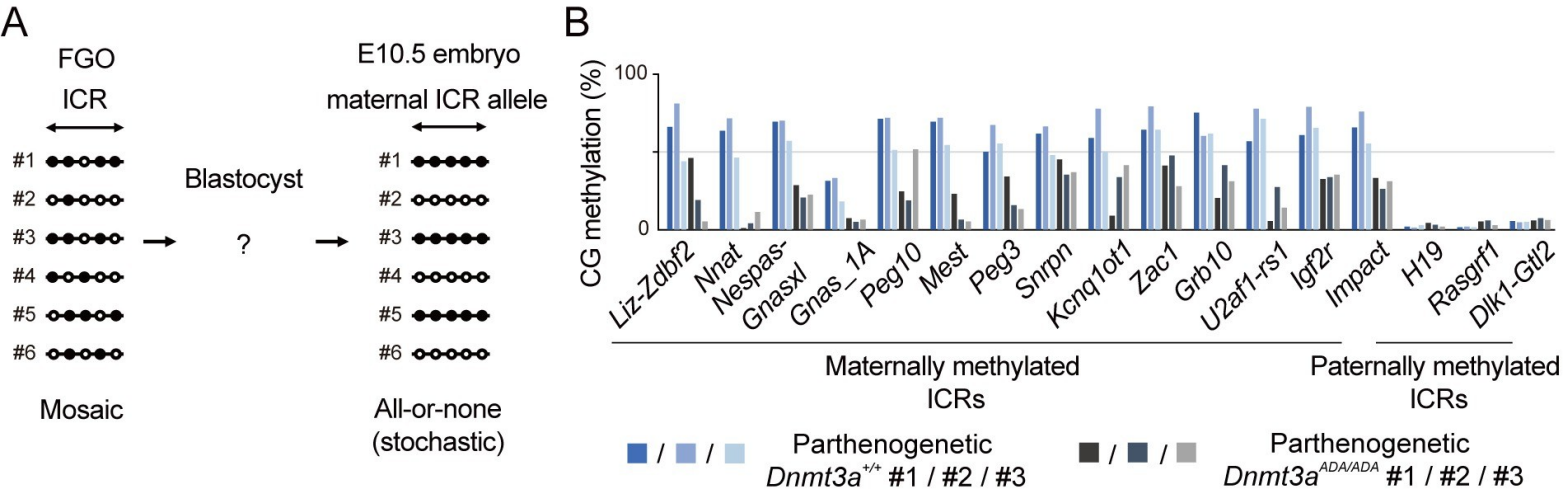
Stochastic loss of ICR methylation occurs during development. (*A*) A model showing the conversion of a partial loss of CG methylation in oocytes to either full or no methylation in embryos. A hypothetical maternally methylated ICR containing five CG sites is shown. Filled and open circles indicate methylated and unmethylated CG sites, respectively. Each ID indicates a single oocyte or embryo. (*B*) CG methylation levels at maternally and paternally methylated ICRs in individual parthenogenetic blastocysts derived from oocytes of indicated genotypes.

The frequent occurrence of almost fully methylated and almost fully unmethylated ICRs in E10.5 embryos (Fig 4D) can in principle originate from oocytes or arise during development after fertilization. However, considering the methylation distribution of the WGBS reads in oocytes (Fig 3A), their mosaic partial methylation patterns (S2 Fig), and the size of the maternally methylated ICRs (0.7–6.7 kb, mean 3.0 kb), the occurrence of ICRs with such extreme methylation levels is unlikely in oocytes. Our data thus suggest that the partial CG methylation at each ICR is frequently converted to either full or no methylation during development in a stochastic manner (Fig 6A).

To examine whether the conversion occurs before or after implantation, we derived diploid parthenogenetic blastocysts possessing only maternal genomes from ovulated wild-type and *Dnmt3a*^*ADA/ADA*^ oocytes (see Materials and methods) and performed WGBS (S3 Table). The advantage of using parthenogenetic blastocysts is that, unlike SNP-based analysis, all WGBS reads obtained from a small number of cells could be used to examine CG methylation of the maternal genome. While parthenogenetic blastocysts from wild-type oocytes showed 45%-80% CG methylation at the ICRs, recapitulating the methylation levels in normal blastocysts [37], those from *Dnmt3a*^*ADA/ADA*^ oocytes showed less than 50% methylation, with some ICRs showing near complete methylation loss (Fig 6B). This suggests that the stochastic loss occurs, or at least initiates, before implantation but that the regaining occurs after implantation, most likely during the *de novo* methylation phase.

## Discussion

Our study revealed an important biological function of the ADD domain of DNMT3A in somatic cells and oocytes. The impact of the *Dnmt3a*^*ADA*^ mutation in somatic cells was manifested as the dwarf phenotype of homozygous mice. This contrasts with the overgrowth phenotype of Tatton-Brown-Rahman syndrome patients with mutations in this domain [22]. Although the cause of this discrepancy is currently unknown, it is noteworthy that mutations in the PWWP domain of human DNMT3A can lead to both overgrowth (Tatton-Brown-Rahman syndrome) and microcephalic dwarfism [8,22]. The homozygous females also had delivery problems, but embryos from such females were lost at various stages even in healthy foster mothers, suggesting that homozygous oocytes, in most of the cases, cannot support normal development. This is most likely due to the partial loss of DNA methylation in such oocytes and stochastic loss of maternal imprinting in derived embryos (see below).

Second, our study suggested a new molecular function for the ADD domain: in addition to acting as an activation switch and excluding H3K4me2/3-marked regions from methylation, it likely contributes to processive CG methylation. Perhaps the ADD domain tethers DNMT3A to chromatin unmethylated at H3K4 and allows the enzyme to act on consecutive CG sites in a processive manner (S6 Fig). This explains why DNMT3A, which on its own does not show processivity [38], can perform processive CG methylation. The role of the ADD domain in non-CG methylation can also be explained in this context. While we note that the model needs to be tested preferably by an *in vitro* methylation assay with reconstituted nucleosomes, such an assay system is not yet in our hands: we therefore leave it for a future study. The fact that DNMT3L, an interacting partner of DNMT3A, also has an ADD domain [12] suggests the functional importance of this domain in the DNMT3A/DNMT3L complex. In this regard, it is interesting that the PWWP domain of DNMT3A, which recognizes histone H3K36me2/3 [5–7,10], only plays a role in limiting ectopic CG methylation [8,9,11]. We thus speculate that, while both ADD and PWWP domains recognize specific modification states of the histone H3 tail, the former contributes to more efficient enzymatic activity and the latter to higher specificity.

Lastly, we found a stochastic loss of maternal imprinting in embryos derived from homozygous oocytes: the partial methylation loss at the maternally methylated ICRs in oocytes was frequently converted to either complete loss or full recovery of CG methylation in a stochastic manner during development. Our study on parthenogenetic embryos derived from homozygous oocytes revealed that the stochastic loss occurs before implantation and the regaining occurs after implantation. Thus, it appears that the conversion of the partial ICR methylation to an all-or-none pattern follows the global wave of demethylation in pre-implantation embryos and subsequent *de novo* methylation in early post-implantation embryos. Although the determinant for gain or loss is currently unknown, methylation states at some key CG sites, such as those recognized by ZFP57, could affect the entire ICR [39,40]. ZFP57 is a nuclear factor that binds to its target sequences within the ICRs [40], only when the internal CG site is methylated, and maintains their methylated state against the demethylation wave in pre-implantation embryos [39]. Consistent with this idea, a recent study by epigenome editing showed that artificial gain and loss of CG methylation at ZFP57 binding sites respectively alter the imprinting state of the ICR and whole imprinted gene cluster in embryonic stem cells, which then persists through neuronal differentiation [41]. The stochastic imprinting loss poses a model to study how partial epigenetic changes induced in germ cells by, for example, environmental factors [42,43] can be transmitted to the successive generations and affect their phenotype.

In conclusion, our study reveals that the DNMT3A ADD domain contributes to efficient, and likely processive, *de novo* DNA methylation, maternal imprinting, and normal embryonic development. Since mutations in this domain are found in congenital growth disorders and hematologic malignancy in humans [19–22], our findings provide a molecular basis for understanding these diseases. The study also identifies a case to study how stochastic epigenetic inheritance can occur in mammalian reproduction.

## Materials and methods

### Ethics statement

All animal experiments were performed under the ethical guidelines of Kyushu University and the protocols were approved by the Institutional Animal Care and Use Committee of Kyushu University (approval number: A22-087-1). Animals were group-housed in a specific-pathogen-free facility under the standard housing condition (12-h light/dark cycle with lights on at 8 am, temperature 20–22°C with ad libitum access to water and food).

### Mutant mice

*Dnmt3a*^*ADA*^ mutant mice were generated using a CRISPR-Cas9 method previously described by Inui et al. [44]. In brief, pX330 plasmid encoding Cas9 and guide RNA (gRNA) and single-stranded donor oligonucleotides (ssODN) were microinjected into mouse zygotes derived from (C57BL/6N× C3H/HeN) F1 females crossed with C57BL/6J males. The gRNA and ssODN sequences were described in S9 Table. The injected zygotes were transferred into the oviducts of pseudo-pregnant ICR females. Pups were genotyped by polymerase chain reaction (PCR) based Sanger sequencing. The primers used for genotyping are listed in S9 Table. A male carrying the expected *Dnmt3a*^*ADA*^ mutation was crossed with C57BL/6J females and offspring carrying the mutation were backcrossed to C57BL/6J at least five times.

### Fertility testing

Female mice at 8 to 24 weeks old were placed with a C57BL/6J or a JF1 male in the same cage, and vaginal plugs were checked the next morning.

### Oocyte collection, IVF and two-cell embryo transfer

GOs were harvested by sequential digestion of P12 ovaries in 1 mg/ml collagenase (034–10533, Wako Pure Chemical), 0.25% trypsin-EDTA (35555–54, Nacalai Tesque), and 0.5% protease type XIV (P5147, Sigma-Aldrich). FGOs were harvested from adult ovaries (10 to 12 weeks old) by needle puncture and pooled in M2 medium (Sigma). To obtain MII oocytes, superovulation was performed by injecting females at 4 to 5 weeks old sequentially with inhibin antiserum (CARD HyperOva, Kyudo) and human chorionic gonadotropin. Cumulus-oocyte complexes were collected from the ampulla of oviducts and fertilized with C57BL/6J sperm in modified human tubal fluid (CARD mHTF, Kyudo). Cumulus cells were removed by capillary washing, and fertilized eggs were cultured in EmbryoMax KSOM Medium (1X) w/ 1/2 Amino Acids (Merck Millipore) at 37°C and 5% CO_2_. After overnight culture, two-cell embryos were transferred into the oviducts of pseudo-pregnant ICR females.

### Parthenogenetic blastocysts

Production of parthenogenetic blastocysts was performed as reported by Kishigami and Wakayama [45]. To obtain MII oocytes, female mice (10 to 15 weeks old) were injected sequentially with pregnant mare serum gonadotropin and human chorionic gonadotropin. Cumulus-oocyte complexes were collected from the ampulla of oviducts and treated with 10 μg/ml hyaluronidase in KSOM medium to remove cumulus cells. After at least 20 minutes culture at 37°C with 5% CO_2_, the MII oocytes were activated by 5 mM SrCl_2_ in 2 mM ethylene glycol-bis(β-aminoethylether)-N,N,N’,N’-tetraacetic acid (EGTA)-containing KSOM medium in the presence of 5 μg/ml cytochalasin B for 6 h and cultured in KSOM medium at 37°C and 5% CO_2_ for 5 days (120 h).

### Embryo dissection and RNA/DNA extraction

Embryos and placentas were dissected from the uteri of female mice crossed with a JF1 male and stored in phosphate-buffered saline. DNA and total RNA were extracted from embryos using AllPrep DNA/RNA Mini kit (QIAGEN).

### WGBS

Pooled (150–250) P12 GOs, pooled (100–250) FGOs, and single parthenogenetic blastocysts were directly spiked with 20, 20, and 2 pg unmethylated lambda phage DNA (Promega), respectively, and subjected to bisulfite treatment and library construction by the post-bisulfite adaptor tagging method [46]. WGBS libraries from FGOs and blastocysts were amplified with KAPA library amplification kit (KAPA) for 4 cycles. Libraries from whole E10.5 embryos were prepared using 200 ng DNA spiked with 2 ng lambda phage DNA by the same method. Concentrations of WGBS libraries were determined by quantitative PCR (qPCR) using a KAPA library quantification kit (KAPA). WGBS libraries were sequenced on NovaSeq 6000 (Illumina) (P12 GOs, NVCS v1.7.5 and RTA v3.4.4; FGOs Rep1, NVCS v1.6.0 and RTA v3.4.4; FGOs Rep2, blastocysts, and E10.5 embryos, NVCS v1.7.0 and RTA v3.4.4) using a NovaSeq 6000 S1 Reagent Kit (Illumina) (FGOs: Rep1) and NovaSeq 6000 SP Reagent Kit (Illumina) (P12 GOs, FGOs: Rep2, blastocysts, E10.5 embryos) to generate 108-nucleotide single-end reads. Low-quality bases and adaptor sequences were removed from total reads using Trim-Galore v0.5.0 (Babraham Institute). The reads were mapped to mouse genome mm10 using Bismark v0.20.0 [47]. Methylation data at CG and non-CG sites covered with 4–100 reads were extracted for subsequent analyses. Windows 10 kb in size with < 5 informative CG and non-CG sites were excluded. For allelic-specific analyses, reads were mapped to the N-masked mm10 genome using published SNP data of JF1 [23]. Allelic reads were selected using SNPsplit v0.3.2 [48] and methylation data at CG sites covered with 1–100 selected reads were extracted for subsequent analyses.

### WGBS single read analysis

Individual WGBS reads containing the same number of CGs and the same number of methylated CGs (thus the same CG methylation ratio) were selected from the entire genome of wild-type GOs at P10 and P12, and wild-type and homozygous FGOs (6 methylation ratio, in the range of 41.7% (5/12) and 54.5% (6/11) CG methylation). We then determined the longest stretches of methylated CGs (or the largest number of consecutively methylated CGs) in individual reads and compared the mean maximum length between the different samples (genotype, developmental stage, expected) for each methylation ratio. Kullback–Leibler divergences of the observed distributions of the longest stretches of methylated CGs per read from the expected distribution were calculated for each methylation ratios using R.

### RNA-seq

RNA-seq libraries were constructed from 0.5 μg total RNA prepared from whole E10.5 embryos using the NEBNext polyA isolation module (NEB#7490S) and NEBNext Ultra II Directional RNA Library Prep Kit for Illumina (NEB#7760). Concentrations of RNA-Seq libraries were determined by qPCR using a KAPA library quantification kit (KAPA). RNA-seq libraries were subjected to paired-end sequencing (53 nucleotides each) on NovaSeq 6000 (Illumina) (NVCS v1.6.0/v1.7.0 and RTA v3.4.4) using a NovaSeq 6000 SP Reagent Kit (Illumina). Raw FASTQ files were trimmed with Trim-Galore v0.5.0 (Babraham Institute), and rRNA sequences were filtered out with Bowtie2-2.3.5.1 [49]. The trimmed reads were mapped to the mouse genome mm10 by HISAT2-2.2.0 [50]. Transcripts were assembled by StringTie-2.1.5 [51]. Genes with FDR < 0.05 in all replicates were defined as differentially expressed genes. Hierarchical clustering of the gene expression profiles was performed using scikit-learn v0.23.1 [52] and python 3.6.8 (https://www.python.org). The CpG island track of mm10 was downloaded from the UCSC Genome Browser. The ICR coordinates were adapted for mm10 [53–56].

### Western blotting

Fifty FGOs were boiled in sample buffer (125 mM Tris-Cl, 2% SDS, 20% glycerol, 0.6 mM bromophenol blue, 9.9% 2-mercaptoethanol, pH 6.8) at 95°C for 3 minutes. Proteins were subjected to 8% sodium dodecyl sulfate-polyacrylamide gel electrophoresis (SDS-PAGE) for 90 minutes at 100 V and transferred to a polyvinylidene difluoride membrane by wet blotting. The membrane was blocked with 3% skimmed milk in wash buffer (2 mM Tris-Cl, 0.02% NaCl, 0.05% Tween20, pH 8.0) for 30 minutes at room temperature and incubated with primary antibodies against DNMT3A (1:100, IMG-268, IMG) and β-actin (1:500, sc-69879, Santa Cruz) in wash buffer overnight at 4°C. Horseradish peroxidase (HRP)-conjugated mouse IgG antibody (1:20000, ab6789, Abcam) was used as the secondary antibody. The membrane was incubated with Chemi-Lumi One Ultra (Nacalai Tesque), and the resulting chemiluminescence signals were detected on an ImageQuant LAS4000 mini (Cytiva).

### Protein synthesis and purification

Complementary DNAs (cDNAs) were generated using 1 μg total RNA from the ovaries as templates using FastGene RNA Premium kit (NIPPON Genetics). cDNA fragments encoding either wild-type or mutated ADD domain (residues 472–605) were amplified by PCR and cloned into pGEX6p-1 vector. The primers used for cloning are listed in S9 Table. The glutathione S-transferase fusion proteins were expressed in *Escherichia coli* strain Rosetta. The cultures with optical density of 0.6–0.8 were added with 0.2 mM isopropyl-β-D-thiogalactopyranoside and incubated at 20°C for 15 hours. After sonication, the supernatant of cell lysate was mixed with Glutathione Sepharose 4 Fast Flow (Cytiva) and applied onto columns for gravity-flow chromatography (Polypropylene Columns, QIAGEN). Then, the fusion proteins were recovered with elution buffer (50 mM Tris-HCl, 10 mM glutathione, pH 8.0). The eluted proteins were purified by Amicon Ultra Centrifugal Filters and subjected to SDS-PAGE followed by Coomassie blue staining for product size confirmation.

### Pulldown assay

For histone H3 peptide pull-down assay, 0.5 μg biotinylated histone H3 peptides (residues 1–21) were incubated with 10 μl streptavidin beads in binding buffer (20 mM Tris-Cl, 250 mM NaCl, 0.01% 2-mercaptoethanol, 5% glycerol and 0.1% Triton X-100, pH 8.0) at 4°C for 30 minutes. Then, 7.5 μg ADD fusion proteins were added into the mixture and incubated at 4°C for 30 minutes. After washing three times with binding buffer, 100% bound and 2.5% unbound proteins were subjected to SDS-PAGE followed by SYPRO Ruby (SYPRO RUBY Protein Gel Stain, Sigma) staining. Signals were detected on Typhoon TRIO (Cytiva) and quantified by Image Quant 5.2.

### Statistical analyses and graph generation

Statistical analyses and graph generation were performed using R v4.3.0 (https://www.r-project.org) and Excel 2016.

## Supporting information

S1 Fig Phenotype of *Dnmt3a*^*ADA*^ mice. (*A*) Representative images of histone H3 peptide pull-down assay. Recombinant proteins containing glutathione S-transferase (GST) fused with wild-type (GST-ADD^WT^) or mutant ADD domain (GST-ADD^ADA^) were incubated with biotinylated histone H3 peptides (residues 1–21) that were un-, mono-, di- or tri-methylated at lysine-4 (H3K4me0/1/2/3) and immobilized onto streptavidin beads (see Materials and methods). Bound (B) and unbound (U) proteins (100% and 2.5%, respectively) were subjected to SDS–polyacrylamide gel electrophoresis and stained with SYPRO Ruby. A negative control (NC) indicates an assay with no histone peptide. (*B*) Proportion of bound per total (= bound plus unbound) quantified by Image Quant 5.2 (n = 3). Red bars show mean. *P* values were determined by the two-sample *t*-test (*P < 0.05). (*C*) Genotype analysis of pups obtained by intercrossing *Dnmt3a*^*ADA/*+^ mice. One hundred and six pups from 15 litters were genotyped. (*D*) Body weight changes after weaning (left) and body weight distribution at postnatal day 49 (P49) (right). *P* values were determined by the two-sample *t*-test (**P < 0.001; *P < 0.05). (*E*) Gross morphology of mice representative of indicated genotype. (*F*) Body size distributions of E10.5 embryos resulting from indicated crosses. *P* values were determined by the two-sample *t*-test (*P < 0.05).
(PDF)

S2 Fig Mosaic loss of CG methylation at the *Impact* ICR in homozygous FGOs. WGBS reads (blue) from wild-type and homozygous FGOs are aligned to the CpG island portion (green) of the *Impact* ICR, with methylated CG sites shown by black boxes and unmethylated ones by white boxes.
(PDF)

S3 Fig Morphology of individual embryos used for WGBS and RNA-seq. Females were crossed with JF1 males, and embryos were obtained at E10.5. Maternal genotypes are indicated. Note that mat-*Dnmt3a*^*ADA/ADA*^ embryos #16, #18, and #20 had retarded growth. All embryos were used for RNA-seq and five of them (#1, 2, 12, 13, and 16) were used for WGBS. Scale bar = 1mm.
(PDF)

S4 Fig Global CG methylation levels in embryos. Violin plots show the distributions of CG methylation levels of 10-kb genomic bins in E10.5 embryos (parental alleles not distinguished). The maternal genotype and embryo ID are indicated. The number above each plot indicates the global CG methylation level.
(PDF)

S5 Fig The altered expression of imprinted genes is correlated with the loss of ICR methylation. (*A*) Dot plots showing the expression levels of the 45 maternally imprinted genes in individual E10.5 embryos (S8 Table). Each dot represents the transcripts per kilobase million (TPM) value of the indicated gene in one of the twenty embryos. TPM values from the same genotype are shown in the same color. Horizontal bars represent the mean TPM values of the indicated gene for the respective genotypes. (*B*) Dot plots showing the degrees of expression change of 40 maternally imprinted genes in respective embryos relative to the mean expression value of the individual genes in embryos derived from wild-type females (#1-#4). Five maternally imprinted genes showing very low expression levels (TPM < 1) in all embryos were excluded from the analysis. The data clearly shows the stochastic misregulation of these genes in mat-*Dnmt3a*^*ADA/ADA*^ embryos. (*C*) Allelic CG methylation states of the maternally methylated ICRs and allelic expression states of the linked genes are shown for imprinted gene clusters other than those shown in Fig 5D SNP-based allele-specific analyses were conducted. The *H19* cluster is regulated by a paternally methylated ICR (control). Red triangles indicate stochastic CG methylation loss and altered expression. CPM, count per million.
(PDF)

S6 Fig A model of DNMT3A ADD domain-mediated processive *de novo* methylation in mouse FGOs. The ADD domain tethers DNMT3A to target chromatin through its interaction with H3K4me0 for processive DNA methylation (left); disruption of this interaction results in non-processive mosaic methylation in homozygous FGOs (right). Filled and open circles indicate methylated and unmethylated CG sites, respectively.
(PDF)

S1 Table Summary of IVF and 2-cell embryo transfer. (PDF)

S2 Table Developmental analysis of embryos derived by crossing females of indicated genotypes with JF1 males. (PDF)

S3 Table Summary of WGBS. (PDF)

S4 Table Mean of the maximum lengths of methylated CG stretch per read for different methylation ratios. (PDF)

S5 Table Kullback–Leibler divergences of the observed distributions of the maximum lengths of methylated CG stretch per read from the expected distribution for different methylation ratios. (PDF)

S6 Table Summary of RNA-seq. (PDF)

S7 Table List of differentially expressed genes. (PDF)

S8 Table List of transcripts per kilobase million (TPM) values of genes regulated by maternally methylated ICRs in individual embryos. (PDF)

S9 Table Oligonucleotide primers used in this study. (PDF)
