# Supplementary material for: The DNMT3A ADD domain is required for efficient de novo DNA methylation and maternal imprinting in mouse oocytes
Source: PLoS Genet. 2023 Aug 1;19(8):e1010855. doi: 10.1371/journal.pgen.1010855 (PMC10393158; doi:10.1371/journal.pgen.1010855)

S1 Fig

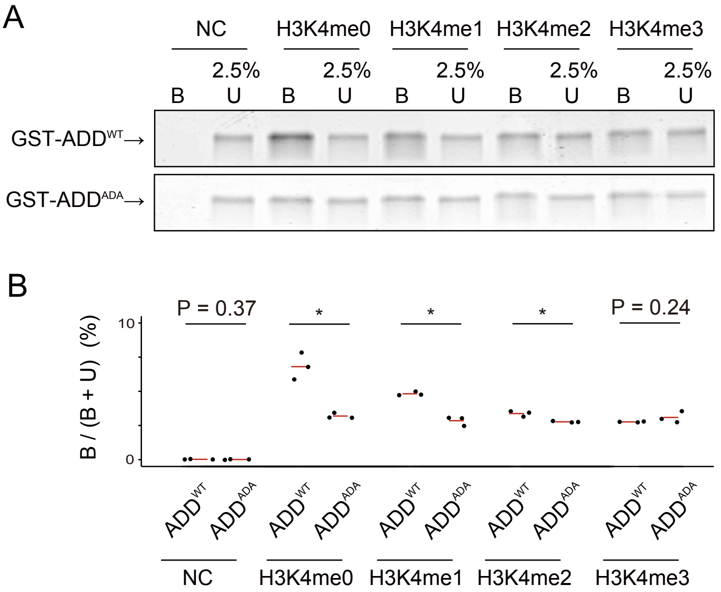

C

Genotype	*Dnmt3a*$^{+/+}$	*Dnmt3a*$^{ADA/+}$	*Dnmt3a*$^{ADA/ADA}$	Total
Number of observed pups	26	55	25	106
Proportion (%)	24.5	51.9	23.6	100
Expected proportion (%)	25	50	25	100

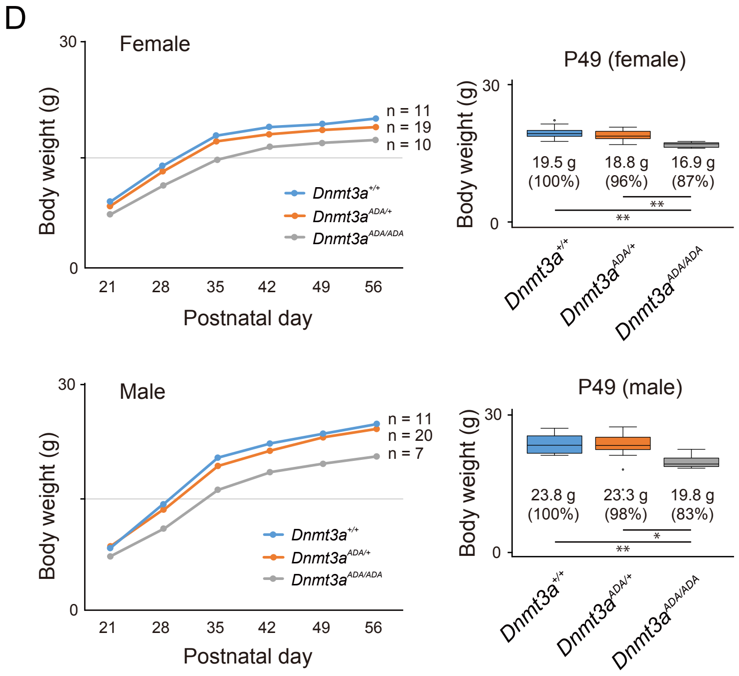

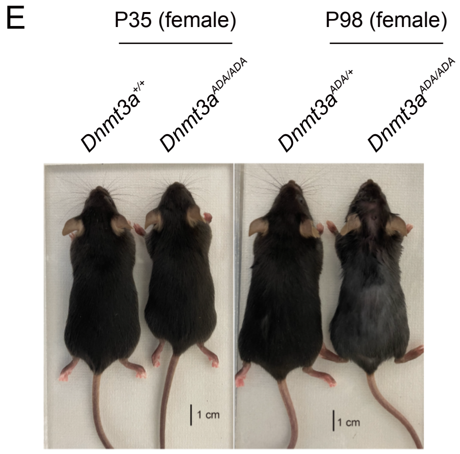

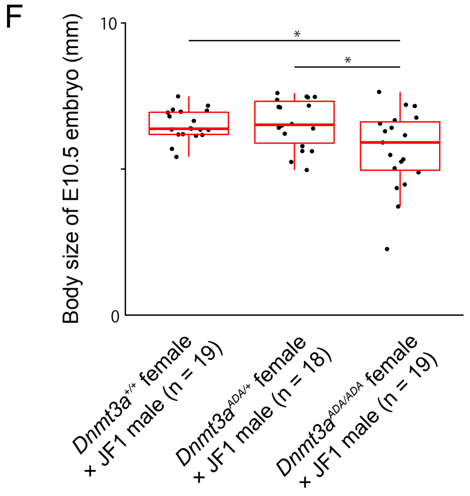

Supplement: S1 Fig — (A) Representative images of histone H3 peptide pull-down assay. Recombinant proteins containing glutathione S-transferase (GST) fused with wild-type (GST-ADDWT) or mutant ADD domain (GST-ADDADA) were incubated with biotinylated histone H3 peptides (residues 1–21) that were un-, mono-, di- or tri-methylated at lysine-4 (H3K4me0/1/2/3) and immobilized onto streptavidin beads (see Materials and methods). Bound (B) and unbound (U) proteins (100% and 2.5%, respectively) were subjected to SDS–polyacrylamide gel electrophoresis and stained with SYPRO Ruby. A negative control (NC) indicates an assay with no histone peptide. (B) Proportion of bound per total (= bound plus unbound) quantified by Image Quant 5.2 (n = 3). Red bars show mean. P values were determined by the two-sample t-test (*P < 0.05). (C) Genotype analysis of pups obtained by intercrossing Dnmt3aADA/+ mice. One hundred and six pups from 15 litters were genotyped. (D) Body weight changes after weaning (left) and body weight distribution at postnatal day 49 (P49) (right). P values were determined by the two-sample t-test (**P < 0.001; *P < 0.05). (E) Gross morphology of mice representative of indicated genotype. (F) Body size distributions of E10.5 embryos resulting from indicated crosses. P values were determined by the two-sample t-test (*P < 0.05). (PDF) [file pgen.1010855.s001.pdf]
