# Supplementary figures and images for: The DNMT3A ADD domain is required for efficient de novo DNA methylation and maternal imprinting in mouse oocytes
Source: PLoS Genet. 2023 Aug 1;19(8):e1010855. doi: 10.1371/journal.pgen.1010855 (PMC10393158; doi:10.1371/journal.pgen.1010855)

S2 Fig

chr18: 12,973,302-12,973,796

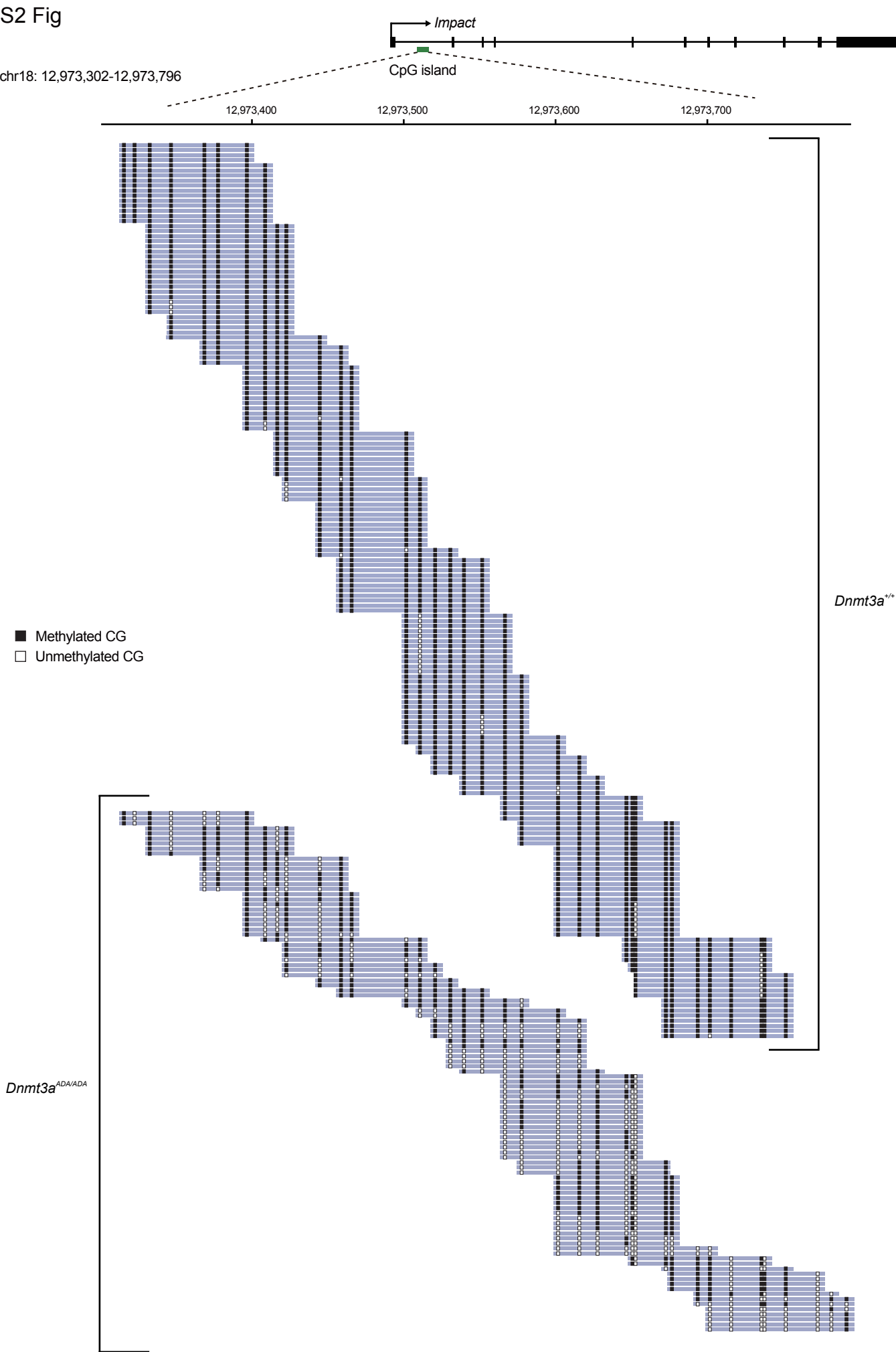

Supplement: S2 Fig — WGBS reads (blue) from wild-type and homozygous FGOs are aligned to the CpG island portion (green) of the Impact ICR, with methylated CG sites shown by black boxes and unmethylated ones by white boxes. (PDF) [file pgen.1010855.s002.pdf]

S3 Fig

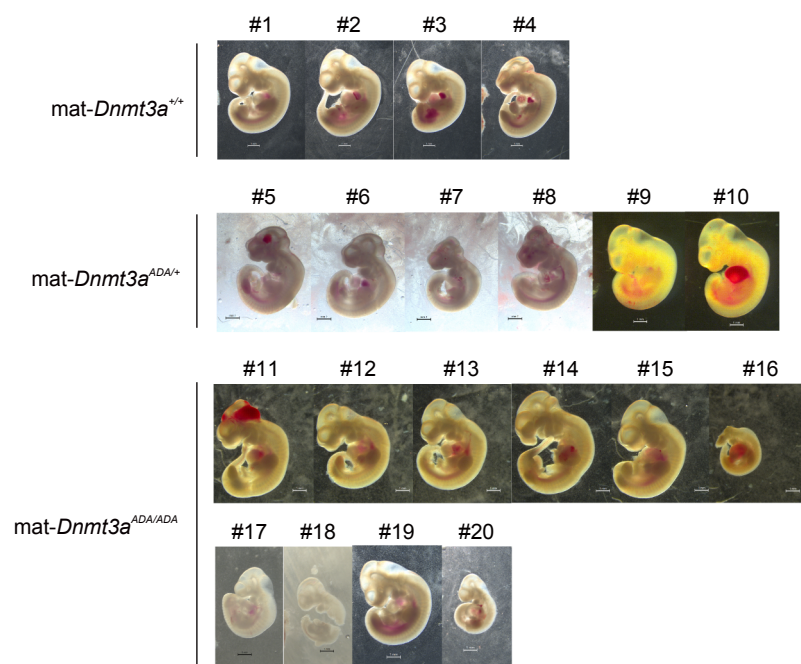

Supplement: S3 Fig — Females were crossed with JF1 males, and embryos were obtained at E10.5. Maternal genotypes are indicated. Note that mat-Dnmt3aADA/ADA embryos #16, #18, and #20 had retarded growth. All embryos were used for RNA-seq and five of them (#1, 2, 12, 13, and 16) were used for WGBS. Scale bar = 1mm. (PDF) [file pgen.1010855.s003.pdf]

S4 Fig

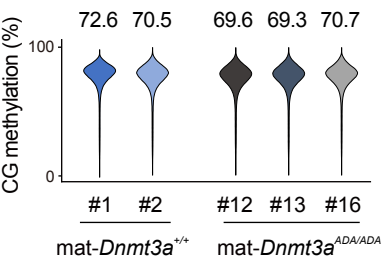

Supplement: S4 Fig — Violin plots show the distributions of CG methylation levels of 10-kb genomic bins in E10.5 embryos (parental alleles not distinguished). The maternal genotype and embryo ID are indicated. The number above each plot indicates the global CG methylation level. (PDF) [file pgen.1010855.s004.pdf]

S5 Fig

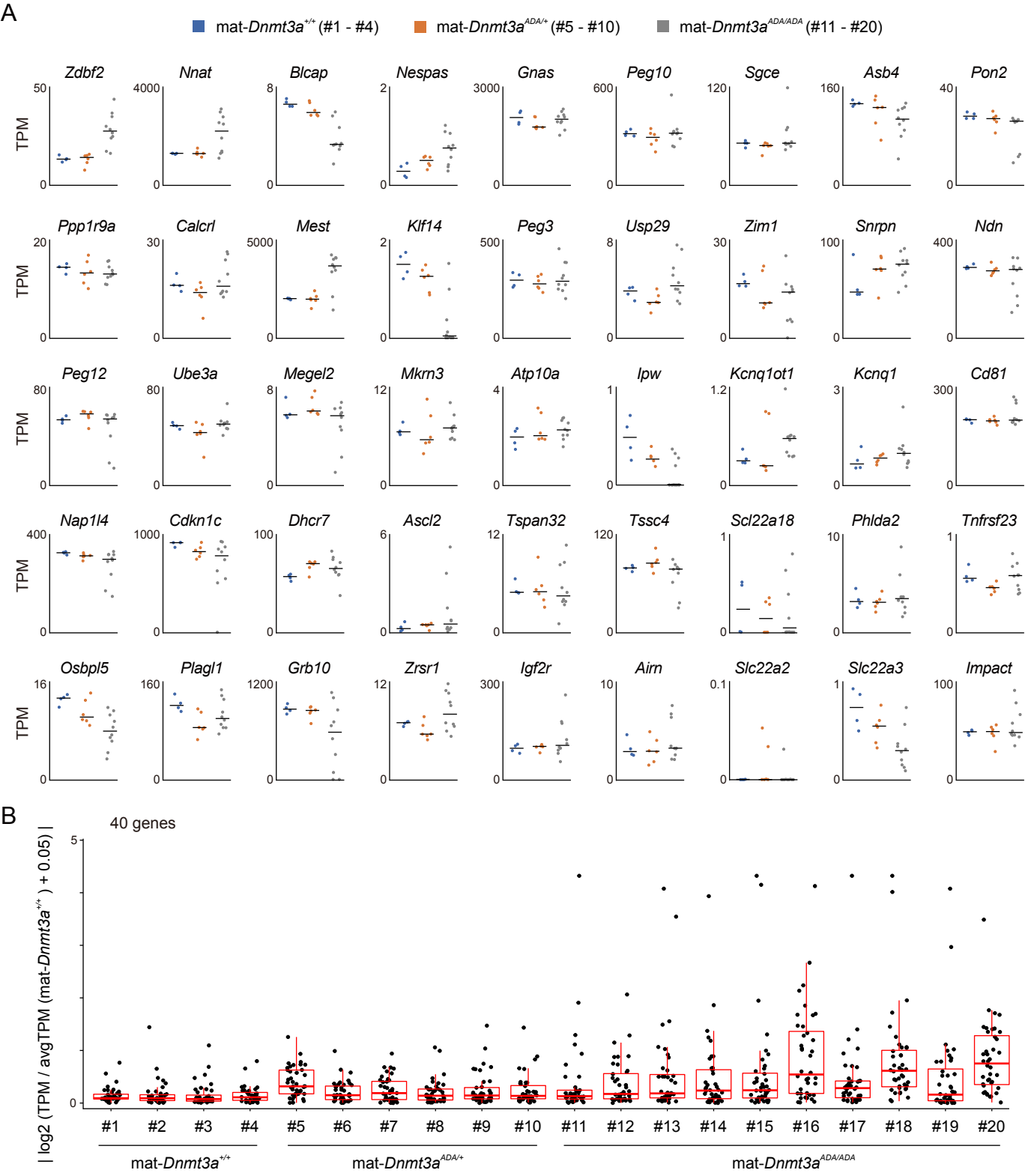

S5 Fig

C

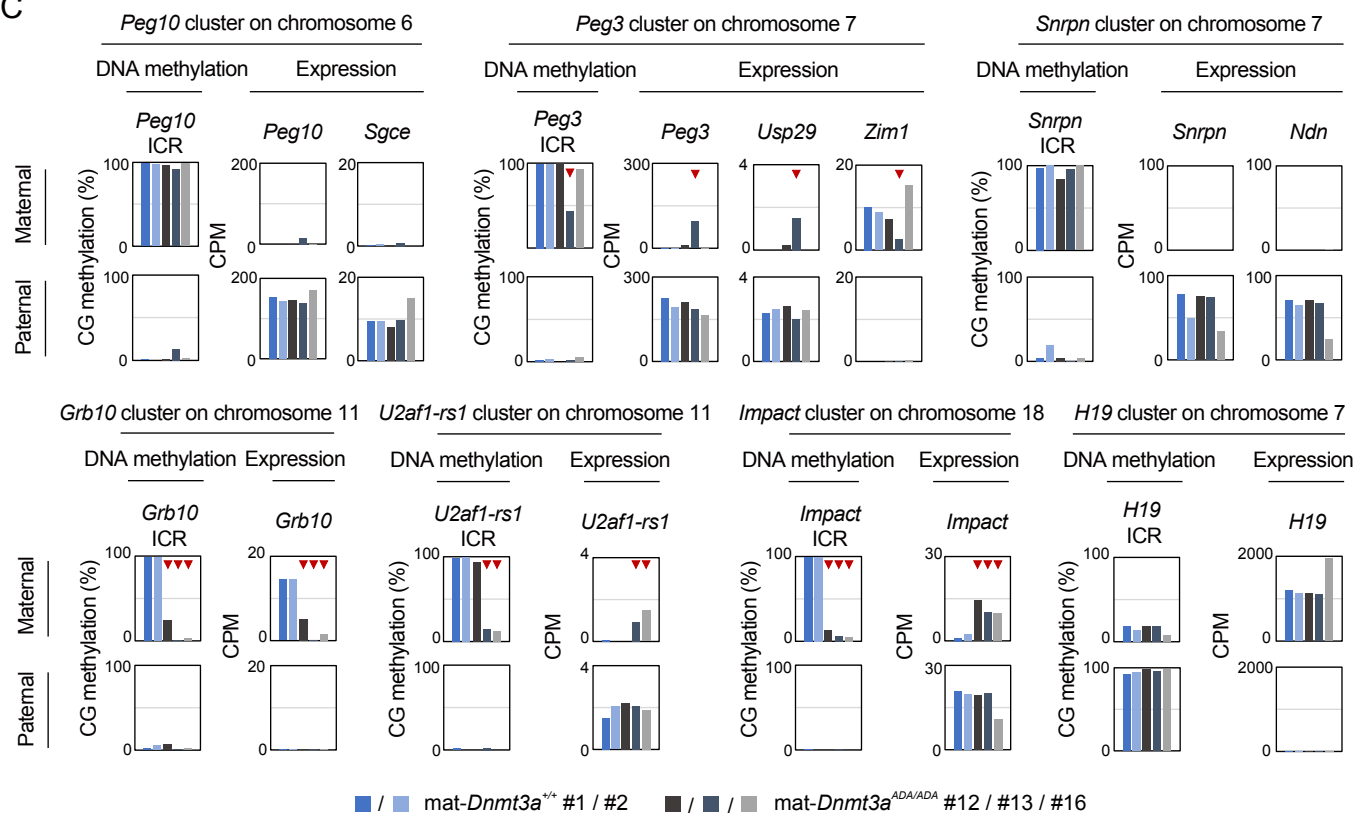

Supplement: S5 Fig — (A) Dot plots showing the expression levels of the 45 maternally imprinted genes in individual E10.5 embryos (S8 Table). Each dot represents the transcripts per kilobase million (TPM) value of the indicated gene in one of the twenty embryos. TPM values from the same genotype are shown in the same color. Horizontal bars represent the mean TPM values of the indicated gene for the respective genotypes. (B) Dot plots showing the degrees of expression change of 40 maternally imprinted genes in respective embryos relative to the mean expression value of the individual genes in embryos derived from wild-type females (#1-#4). Five maternally imprinted genes showing very low expression levels (TPM < 1) in all embryos were excluded from the analysis. The data clearly shows the stochastic misregulation of these genes in mat-Dnmt3aADA/ADA embryos. (C) Allelic CG methylation states of the maternally methylated ICRs and allelic expression states of the linked genes are shown for imprinted gene clusters other than those shown in Fig 5D SNP-based allele-specific analyses were conducted. The H19 cluster is regulated by a paternally methylated ICR (control). Red triangles indicate stochastic CG methylation loss and altered expression. CPM, count per million. (PDF) [file pgen.1010855.s005.pdf]

S6 Fig

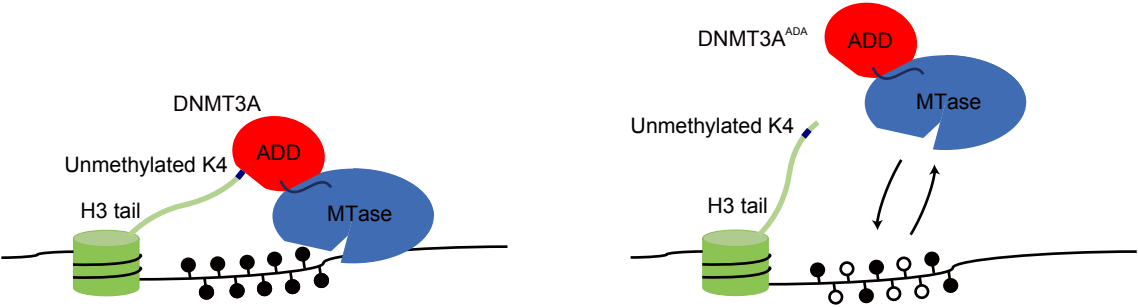

Supplement: S6 Fig — The ADD domain tethers DNMT3A to target chromatin through its interaction with H3K4me0 for processive DNA methylation (left); disruption of this interaction results in non-processive mosaic methylation in homozygous FGOs (right). Filled and open circles indicate methylated and unmethylated CG sites, respectively. (PDF) [file pgen.1010855.s006.pdf]
