# Supplementary material for: The DNMT3A ADD domain is required for efficient de novo DNA methylation and maternal imprinting in mouse oocytes
Source: PLoS Genet. 2023 Aug 1;19(8):e1010855. doi: 10.1371/journal.pgen.1010855 (PMC10393158; doi:10.1371/journal.pgen.1010855)
Supplement: S1 Table — (PDF) [file pgen.1010855.s007.pdf]

S1 Table: Summary of IVF and 2-cell embryo transfer.

Genotype of oocyte	Litter number	Number of transferred embryos	Number of live pups	Number of stillborn pups	Proportion of live among transferred (%)
Dnmt3a$^{+/+}$	#1	20	4	0	26.7
	#2	20	0	2	
	#3	20	10	0	
Dnmt3a$^{ADA/+}$	#1	20	5	0	25.0
	#2	20	5	0	
Dnmt3a$^{ADA/ADA}$	#1	20	0	1	3.3
	#2	20	2	0	
	#3	20	0	1	
**Dnmt3a*$^{+/+}$ + *Dnmt3a*$^{ADA/ADA}$	#1	12+8	6+0	0	(50.0 + 0.0)

* 2-cell embryos were mixed and transfered, and pups were genotyped.
