# Supplementary material for: The DNMT3A ADD domain is required for efficient de novo DNA methylation and maternal imprinting in mouse oocytes
Source: PLoS Genet. 2023 Aug 1;19(8):e1010855. doi: 10.1371/journal.pgen.1010855 (PMC10393158; doi:10.1371/journal.pgen.1010855)
Supplement: S2 Table — (PDF) [file pgen.1010855.s008.pdf]

S2 Table: Developmental analysis of embryos derived by crossing females of indicated genotypes with JF1 males.

Stage	Genotype of mother	Genotype of embryo	Number of implantation	Number of normal embryos	Number of abnormal embryos	Number of implantation sites	Proportion of normal embryo / implantation (%)
E8.5-9.0	*Dnmt3a*$^{ADA/+}$	*Dnmt3a*$^{+/+}$ or *Dnmt3a*$^{ADA/+}$ (mat-*Dnmt3a*$^{ADA/+}$)	10	10	0	0	100.0
			9	9	0	0	
			11	11	0	0	
			9	9	0	0	
	Dnmt3a$^{ADA/ADA}$	*Dnmt3a*$^{ADA/+}$ (mat-*Dnmt3a*$^{ADA/ADA}$)	7	7	0	0	90.9
			7	6	1	0	
			8	7	1	0	
E10.5	*Dnmt3a*$^{ADA/+}$	*Dnmt3a*$^{+/+}$ or *Dnmt3a*$^{ADA/+}$ (mat-Dnmt3a$^{ADA/ADA}$)	10	10	0	0	100.0
			8	8	0	0	
	Dnmt3a$^{ADA/ADA}$	*Dnmt3a*$^{ADA/+}$ (mat-*Dnmt3a*$^{ADA/+}$)	7	5	2	0	84.2
			7	6	1	0	
			5	5	0	0	
E14.5	*Dnmt3a*$^{ADA/+}$	*Dnmt3a*$^{+/+}$ or *Dnmt3a*$^{ADA/+}$ (mat-*Dnmt3a*$^{ADA/+}$)	9	9	0	0	100.0
			7	7	0	0	
	Dnmt3a$^{ADA/ADA}$	*Dnmt3a*$^{ADA/+}$ (mat-Dnmt3a$^{ADA/ADA}$)	7	3	0	4	50.0
			9	5	0	4	
			9	3	0	6	
			9	6	0	3	
E18.5	*Dnmt3a*$^{ADA/+}$	*Dnmt3a*$^{+/+}$ or *Dnmt3a*$^{ADA/+}$ (mat-*Dnmt3a*$^{ADA/+}$)	9	9	0	0	94.1
			8	7	0	1	
	Dnmt3a$^{ADA/ADA}$	*Dnmt3a*$^{ADA/+}$ (mat-Dnmt3a$^{ADA/ADA}$)	4	3	0	1	52.2
			5	1	0	4	
			6	3	0	3	
			8	5	0	3	
