# Supplementary material for: The DNMT3A ADD domain is required for efficient de novo DNA methylation and maternal imprinting in mouse oocytes
Source: PLoS Genet. 2023 Aug 1;19(8):e1010855. doi: 10.1371/journal.pgen.1010855 (PMC10393158; doi:10.1371/journal.pgen.1010855)
Supplement: S3 Table — (PDF) [file pgen.1010855.s009.pdf]

S3 Table: Summary of WGBS.

Sample		Total reads	Mapped reads	Average depth	Conversion error rate (%)	CG methylation (%)	Non-CG methylation (%)	Pearson's correlation coefficient*
Dnmt3a$^{+/+}$ P12 GOs Rep1		369,162,908	219,832,378		1.02	13.3	1.09	
Dnmt3a$^{+/+}$ P12 GOs Rep2		384,551,418	229,601,761		1.09	12.4	1.21	Rep1 vs Rep2: 0.93
	Total	753,714,326	449,434,139	18.7		12.8	1.15	
Dnmt3a$^{+/+}$ FGOs Rep1		700,855,140	377,060,065		0.58	35.2	3.17	
Dnmt3a$^{+/+}$ FGOs Rep2		419,495,891	252,117,030		0.97	36.3	3.70	Rep1 vs Rep2: 0.99
	Total	1,120,351,031	629,177,096	26.2		35.8	3.38	
Dnmt3a$^{ADA/+}$ FGOs Rep1		680,896,848	295,509,232		0.48	33.6	2.32	
Dnmt3a$^{ADA/+}$ FGOs Rep2		419,495,891	261,765,436		0.69	33.3	2.51	Rep1 vs Rep2: 0.99
	Total	1,100,392,739	557,274,668	21.5		33.8	2.42	
Dnmt3a$^{ADA/ADA}$ FGOs Rep1		683,234,087	318,387,084		0.43	17.6	0.52	
Dnmt3a$^{ADA/ADA}$ FGOs Rep2		346,064,243	213,867,702		0.74	17.5	0.83	Rep1 vs Rep2: 0.98
	Total	1,029,298,330	532,254,787	22.1		17.6	0.65	
Parthenogenetic blastocyst from *Dnmt3a*$^{+/+}$ #1		420,960,713	258,469,878	10.7	1.05	14.6	1.04	#1 vs #2: 0.86
Parthenogenetic blastocyst from *Dnmt3a*$^{+/+}$ #2		364,557,851	221,286,616	9.2	1.17	15.7	1.24	#1 vs #3: 0.85
Parthenogenetic blastocyst from *Dnmt3a*$^{+/+}$ #3		404,249,043	241,336,679	10	0.91	12.6	0.97	#2 vs #3: 0.86
Parthenogenetic blastocyst from *Dnmt3a*$^{ADA/ADA}$ #1		424,551,890	261,523,964	10.9	0.81	12.5	0.94	#1 vs #2: 0.80
Parthenogenetic blastocyst from *Dnmt3a*$^{ADA/ADA}$ #2		420,641,955	256,591,592	10.7	0.94	12.0	1.00	#1 vs #3: 0.80
Parthenogenetic blastocyst from *Dnmt3a*$^{ADA/ADA}$ #3		412,226,709	252,282,745	10.5	1.02	9.9	1.06	#2 vs #3: 0.82
E10.5 mat-*Dnmt3a*$^{+/+}$ embryo #1		398,812,427	223,126,944	9.3	0.58	72.6	0.56	
	Maternal		26,493,091			74.1	0.56	
	Paternal		23,810,185			72.5	0.58	#1 vs #2: 0.93
E10.5 mat-*Dnmt3a*$^{+/+}$ embryo #2		400,142,553	227,280,970	9.5	0.57	70.5	0.63	
	Maternal		27,059,704			73.4	0.56	
	Paternal		24,303,231			71.8	0.58	
E10.5 mat-*Dnmt3a*$^{ADA/ADA}$ embryo #12		347,468,660	197,014,730	8.2	0.58	69.6	0.60	
	Maternal		23,436,275			72.6	0.56	
	Paternal		21,170,961			70.9	0.58	
E10.5 mat-*Dnmt3a*$^{ADA/ADA}$ embryo #13		358,494,020	204,700,085	8.5	0.57	69.3	0.62	#12 vs #13: 0.92
	Maternal		24,910,092			72.2	0.56	#13 vs #16: 0.93
	Paternal		21,886,699			70.7	0.58	#12 vs #16: 0.92
E10.5 mat-*Dnmt3a*$^{ADA/ADA}$ embryo #16		372,291,537	210,344,718	8.7	0.58	70.7	0.65	
	Maternal		25,272,820			74.0	0.58	
	Paternal		22,650,045			72.5	0.60	

* The correlation coefficient of CG methylation levels for each genotype was calculated across all 10-kb bins.
