# Supplementary material for: The DNMT3A ADD domain is required for efficient de novo DNA methylation and maternal imprinting in mouse oocytes
Source: PLoS Genet. 2023 Aug 1;19(8):e1010855. doi: 10.1371/journal.pgen.1010855 (PMC10393158; doi:10.1371/journal.pgen.1010855)
Supplement: S4 Table — (PDF) [file pgen.1010855.s010.pdf]

S4 Table: Mean of the maximum lengths of methylated CG stretch per read for different methylation ratios.

The numbers of CGs and methylated CGs per read	$Dnmt3a^{+/+}$ P10 GOs	$Dnmt3a^{+/+}$ P12 GOs	$Dnmt3a^{+/+}$ FGOs	Expected	$Dnmt3a^{ADA/ADA}$ FGOs
5 mCGs out of 12 CGs	3.1	2.8	2.9	2.4	2.6
5 mCGs out of 11 CGs	3.2	3.1	3.0	2.5	2.8
6 mCGs out of 13 CGs	3.5	3.3	3.4	2.7	3.0
6 mCGs out of 12 CGs	3.9	3.5	3.6	2.9	3.2
7 mCGs out of 13 CGs	4.3	4.0	4.1	3.3	3.7
6 mCGs out of 11 CGs	3.9	3.6	3.7	3.1	3.4
