# Supplementary material for: The DNMT3A ADD domain is required for efficient de novo DNA methylation and maternal imprinting in mouse oocytes
Source: PLoS Genet. 2023 Aug 1;19(8):e1010855. doi: 10.1371/journal.pgen.1010855 (PMC10393158; doi:10.1371/journal.pgen.1010855)
Supplement: S5 Table — (PDF) [file pgen.1010855.s011.pdf]

S5 Table: Kullback–Leibler divergences of the observed distributions of the maximum lengths of methylated CG stretch per read from the expected distribution for different methylation ratios.

The numbers of CGs and methylated CGs per read	Dnmt3a$^{+/+}$ P10 GOs	Dnmt3a$^{+/+}$ P12 GOs	Dnmt3a$^{+/+}$ FGOs	Dnmt3a$^{ADA/ADA}$ FGOs
5 mCGs out of 12 CGs	0.268	0.114	0.16	0.028
5 mCGs out of 11 CGs	0.242	0.177	0.156	0.057
6 mCGs out of 13 CGs	0.323	0.138	0.276	0.059
6 mCGs out of 12 CGs	0.408	0.166	0.194	0.054
7 mCGs out of 13 CGs	0.316	0.175	0.215	0.09
6 mCGs out of 11 CGs	0.255	0.143	0.176	0.049
