# Supplementary material for: The DNMT3A ADD domain is required for efficient de novo DNA methylation and maternal imprinting in mouse oocytes
Source: PLoS Genet. 2023 Aug 1;19(8):e1010855. doi: 10.1371/journal.pgen.1010855 (PMC10393158; doi:10.1371/journal.pgen.1010855)
Supplement: S6 Table — (PDF) [file pgen.1010855.s012.pdf]

S6 Table: Summary of RNA-seq.

Embryo ID	Total reads	Mapped reads		
		Total	Maternal	Paternal
E10.5 mat-*Dnmt3a*$^{+/+}$ embryo #1	59,938,478	53,837,173	6,969,355	6,106,774
E10.5 mat-*Dnmt3a*$^{+/+}$ embryo #2	34,718,959	31,252,565	4,033,946	3,559,685
E10.5 mat-*Dnmt3a*$^{+/+}$ embryo #3	31,888,187	28,582,043	3,770,700	3,186,736
E10.5 mat-*Dnmt3a*$^{+/+}$ embryo #4	41,140,797	35,384,113	4,556,195	4,040,265
E10.5 mat-*Dnmt3a*$^{ADA/+}$ embryo #5	56,299,516	51,067,064	6,769,605	5,816,126
E10.5 mat-*Dnmt3a*$^{ADA/+}$ embryo #6	43,905,758	39,207,737	5,041,511	4,426,472
E10.5 mat-*Dnmt3a*$^{ADA/+}$ embryo #7	47,419,584	42,510,580	5,446,906	4,814,616
E10.5 mat-*Dnmt3a*$^{ADA/+}$ embryo #8	51,066,055	45,704,203	5,884,749	5,202,609
E10.5 mat-*Dnmt3a*$^{ADA/+}$ embryo #9	39,070,227	35,267,200	4,628,781	4,032,444
E10.5 mat-*Dnmt3a*$^{ADA/+}$ embryo #10	40,415,154	36,511,965	4,816,166	4,178,251
E10.5 mat-*Dnmt3a*$^{ADA/ADA}$ embryo #11	26,283,301	23,605,941	3,124,802	2,652,170
E10.5 mat-*Dnmt3a*$^{ADA/ADA}$ embryo #12	36,086,356	32,498,304	4,225,643	3,719,978
E10.5 mat-*Dnmt3a*$^{ADA/ADA}$ embryo #13	36,928,868	33,355,784	4,409,126	3,728,788
E10.5 mat-*Dnmt3a*$^{ADA/ADA}$ embryo #14	43,270,651	38,972,516	5,161,026	4,350,221
E10.5 mat-*Dnmt3a*$^{ADA/ADA}$ embryo #15	25,816,738	23,168,289	3,080,902	2,580,889
E10.5 mat-*Dnmt3a*$^{ADA/ADA}$ embryo #16	26,481,231	23,375,679	3,110,841	2,720,564
E10.5 mat-*Dnmt3a*$^{ADA/ADA}$ embryo #17	31,911,380	28,352,293	3,640,740	3,231,659
E10.5 mat-*Dnmt3a*$^{ADA/ADA}$ embryo #18	24,614,664	21,854,143	2,895,187	2,458,551
E10.5 mat-*Dnmt3a*$^{ADA/ADA}$ embryo #19	21,399,667	18,972,966	2,499,265	2,094,789
E10.5 mat-*Dnmt3a*$^{ADA/ADA}$ embryo #20	32,222,469	28,446,779	3,823,970	3,181,138
