# Supplementary material for: The DNMT3A ADD domain is required for efficient de novo DNA methylation and maternal imprinting in mouse oocytes
Source: PLoS Genet. 2023 Aug 1;19(8):e1010855. doi: 10.1371/journal.pgen.1010855 (PMC10393158; doi:10.1371/journal.pgen.1010855)
Supplement: S7 Table — (PDF) [file pgen.1010855.s013.pdf]

S7 Table: List of differentially expressed genes.

Gene symbol	Change	Log_2 fold change	FDR
Trib3	up	3.73	3.75E-02
Zdbf2	up	1.27	3.95E-04
Mest	up	0.95	5.42E-03
Scd1	up	0.93	1.94E-02
Nnat	up	0.89	1.96E-02
Gm14296	down	-9.26	2.15E-08
Rmrp	down	-2.65	4.73E-03
Blcap	down	-0.86	4.73E-03
