# Supplementary material for: The DNMT3A ADD domain is required for efficient de novo DNA methylation and maternal imprinting in mouse oocytes
Source: PLoS Genet. 2023 Aug 1;19(8):e1010855. doi: 10.1371/journal.pgen.1010855 (PMC10393158; doi:10.1371/journal.pgen.1010855)
Supplement: S8 Table — (PDF) [file pgen.1010855.s014.pdf]

S8 Table: List of transcripts per kilobase million (TPM) values of genes regulated by maternally methylated ICRs in individual embryos.

Gene symbol	E10.5 mat-*Dnmt3a* $^{+/+}$ embryo				E10.5 mat-*Dnmt3a* $^{ADA/+}$ embryo						E10.5 mat-*Dnmt3a* $^{ADA/ADA}$ embryo									
	#1	#2	#3	#4	#5	#6	#7	#8	#9	#10	#11	#12	#13	#14	#15	#16	#17	#18	#19	#20
Airn	4.627	2.623	2.541	3.193	1.883	2.993	1.529	2.891	4.937	4.049	2.132	3.465	3.266	2.578	3.248	6.749	3.202	7.606	2.098	6.404
Asb4	134.001	132.229	128.929	139.308	73.626	127.304	126.546	101.679	145.573	139.139	106.069	134.078	109.844	80.017	122.625	125.338	89.987	99.652	127.309	43.086
Ascl2	0.350	0.104	0.684	0.172	0.437	0.492	0.509	0.141	0.495	0.589	0.787	0.312	0.343	0.251	0.069	1.529	0.741	5.276	0.212	3.657
Atp10a	2.227	1.483	1.749	2.336	1.903	1.894	1.934	2.151	3.176	2.984	2.300	2.757	2.667	2.780	1.894	2.068	2.259	2.563	2.065	1.590
Blcap	64.474	64.474	70.681	67.801	67.435	57.628	56.766	57.109	61.156	68.983	59.924	56.594	33.071	33.697	34.256	32.182	17.468	47.190	29.281	32.721
Calcrl	19.820	16.105	14.329	16.280	6.131	12.659	14.834	13.153	15.518	17.050	13.632	17.421	14.030	14.419	12.611	23.972	19.618	26.464	14.291	25.781
Cd81	200.893	203.619	190.796	201.646	199.884	211.965	195.628	193.891	201.521	184.461	193.904	201.535	196.644	193.480	187.104	261.270	204.860	252.062	198.579	271.201
Cdkn1c	918.896	919.128	921.277	871.504	867.513	813.681	750.639	777.401	921.661	841.817	853.435	825.749	926.409	744.984	931.668	6.570	631.939	552.088	875.570	509.187
Dhcr7	52.500	59.806	56.085	58.583	70.080	71.019	72.360	72.347	66.631	56.889	64.390	74.718	76.914	71.656	66.744	38.004	59.527	61.763	83.269	58.951
Gnas	1868.349	2224.464	1919.567	2269.317	2099.813	1767.277	2090.706	1731.942	1769.805	1793.687	1687.693	1530.107	2153.062	2088.810	2330.324	2256.288	1941.333	1917.238	2107.782	1950.715
Grb10	884.502	851.388	807.501	934.660	691.381	858.275	897.369	819.636	890.579	845.293	858.634	313.175	8.032	505.839	712.177	93.229	665.686	1007.037	8.053	1071.089
Igf2r	109.821	81.997	89.946	104.916	104.313	101.292	108.858	83.346	104.072	102.094	97.589	123.801	100.944	81.875	56.561	260.491	111.690	173.632	91.518	178.873
Impact	50.254	48.336	45.653	51.081	29.109	45.219	56.277	47.512	51.457	53.970	46.991	78.728	66.836	58.010	44.047	46.011	91.486	50.009	45.731	35.000
Ipw	0.250	0.708	0.380	0.591	0.395	0.294	0.245	0.187	0.000	0.283	0.000	0.364	0.275	0.320	0.196	0.000	0.000	0.000	0.000	0.000
Kcnq1	0.540	0.552	1.197	0.776	0.916	0.933	0.635	0.723	0.775	0.974	0.681	1.114	1.140	0.717	1.023	0.555	0.956	1.229	0.760	2.415
Kcnq1ot1	0.282	0.277	0.445	0.324	0.184	0.240	0.230	0.244	0.904	0.881	0.362	0.594	0.354	0.598	0.409	1.182	0.361	0.606	0.556	0.616
Klf14	1.65753	1.20326	1.74552	1.35298	0.92556	1.27822	1.24133	1.37942	1.50012	0.87648	0.000	0.99614	0.05315	0.02294	0.000	1.51738	0.000	0.000	0.11592	0.36371
Magel2	5.843	5.747	5.565	7.226	7.220	5.927	5.837	6.041	7.755	6.172	6.367	6.811	6.299	5.854	5.588	1.082	4.547	4.822	5.977	2.321
Mest	2028.014	1959.547	2016.987	2043.993	1514.698	2135.888	2421.376	1959.904	2026.945	1928.153	3750.583	2114.641	4111.668	3626.699	3468.100	1444.292	4025.354	4088.857	4248.328	3387.717
Mkrn3	6.658	6.223	7.775	6.489	5.904	5.208	3.905	5.314	10.566	8.546	6.972	5.802	6.604	7.281	7.216	5.655	8.653	10.126	7.116	5.598
Nap1l4	325.173	330.897	327.818	317.430	310.173	312.571	326.062	319.215	315.381	293.569	290.630	318.578	319.626	331.755	318.831	148.570	296.253	238.712	303.670	171.398
Ndn	291.982	303.029	284.041	285.634	310.066	270.778	265.148	279.226	284.650	255.320	277.608	293.007	285.937	327.107	328.733	105.257	223.179	172.106	283.221	133.194
Nespas	0.159	0.383	0.189	0.451	0.441	0.572	0.404	0.573	0.592	0.318	1.094	1.221	0.816	1.059	0.420	0.729	0.378	0.571	0.291	0.791
Nnat	1305.490	1305.490	1305.636	1265.089	1270.649	1517.914	1284.948	1152.201	1305.749	1348.776	1305.462	1348.754	2790.776	2557.515	2989.867	3114.341	1397.042	1107.300	1926.823	2498.934
Osbpl5	13.484	13.938	11.896	13.255	14.220	9.672	8.921	9.895	13.017	10.661	11.327	11.826	8.624	9.592	10.613	3.447	7.355	6.354	6.952	4.486
Peg10	326.556	301.843	305.176	343.271	204.712	275.907	311.053	251.333	347.278	318.058	330.345	321.706	314.269	235.496	240.466	327.373	285.927	315.792	343.170	552.800
Peg12	53.061	56.831	54.152	50.857	60.558	56.993	60.232	59.882	55.372	46.488	55.823	57.357	57.607	58.015	55.102	14.194	53.424	40.423	51.067	18.370
Peg3	323.327	269.245	260.615	334.922	233.456	261.568	293.127	255.144	325.994	315.976	272.868	320.127	386.113	306.647	386.897	202.011	274.782	456.318	242.960	250.956
Phlda2	3.001	4.523	3.377	2.573	3.026	3.405	2.110	2.695	3.202	4.241	3.308	2.033	3.672	2.620	3.149	1.637	3.664	3.674	5.909	8.752
Plagl1	125.456	118.168	112.017	140.398	66.021	85.971	85.425	83.246	116.140	110.756	103.017	110.972	137.904	97.927	75.309	131.713	147.353	85.439	90.995	85.617
Pon2	29.917	27.245	29.102	27.272	21.352	25.901	26.831	27.558	28.243	30.215	27.370	26.198	26.361	26.550	26.792	12.626	25.698	11.708	26.119	9.206
Ppp1r9a	14.562	13.051	14.410	15.207	10.124	11.286	13.592	13.040	16.943	15.606	14.544	15.817	12.659	13.227	12.419	13.840	12.986	15.695	10.954	11.034
Sgce	51.488	51.785	45.754	54.780	36.373	48.572	48.718	51.043	51.949	48.028	53.231	50.929	51.750	50.249	47.652	67.889	43.607	70.773	51.394	119.317
Slc22a18	0.521	0.000	0.476	0.000	0.287	0.346	0.000	0.000	0.317	0.000	0.000	0.000	0.153	0.391	0.000	0.000	0.623	0.000	0.793	0.000
Slc22a2	0.000	0.000	0.000	0.000	0.000	0.000	0.000	0.054	0.000	0.034	0.000	0.000	0.000	0.032	0.000	0.000	0.000	0.000	0.000	0.000
Slc22a3	0.872	0.501	0.931	0.608	0.381	0.540	0.328	0.608	0.561	0.763	0.344	0.371	0.286	0.740	0.095	0.568	0.130	0.315	0.206	0.155
Snrpn	49.295	85.339	44.787	44.908	82.059	41.074	71.046	70.050	83.639	69.113	76.447	91.509	46.766	53.052	88.791	64.448	67.404	74.717	79.262	79.277
Tnfrsf23	0.524	0.574	0.538	0.697	0.469	0.389	0.528	0.420	0.470	0.453	0.589	0.589	0.709	0.483	0.442	0.407	0.578	0.807	0.395	0.677
Tspan32	5.039	4.886	6.610	4.895	5.775	4.033	3.135	4.778	5.290	9.256	8.622	4.088	3.833	5.525	3.830	10.729	8.326	1.116	4.952	3.436
Tssc4	79.861	82.529	78.852	74.754	103.630	89.097	81.185	84.296	86.242	72.715	72.992	77.727	83.557	78.925	89.843	30.531	78.167	61.808	80.333	36.694
Ube3a	49.002	48.646	45.885	51.531	23.063	41.637	44.078	42.324	51.656	49.828	50.975	52.522	46.639	49.230	40.850	49.594	51.911	66.798	46.269	50.632
Usp29	4.129	3.623	3.065	4.169	2.082	2.962	2.845	2.918	4.053	3.485	4.011	4.561	5.172	5.757	7.289	3.752	3.050	7.639	2.832	4.038
Zdbf2	13.606	13.049	11.817	15.526	7.664	11.600	15.798	13.447	15.211	14.939	28.521	24.094	24.873	30.010	17.286	36.769	34.972	43.795	26.728	16.311
Zim1	20.085	16.519	16.358	17.743	9.584	10.942	10.760	11.213	22.683	21.237	15.396	16.217	5.130	5.943	0.111	25.913	16.973	5.574	14.318	14.651
Zrsr1	7.157	6.938	6.541	7.103	5.607	4.940	5.640	5.424	6.695	7.763	6.858	7.011	10.633	6.584	5.985	9.527	10.951	5.339	11.786	9.114
