# Supplementary material for: The DNMT3A ADD domain is required for efficient de novo DNA methylation and maternal imprinting in mouse oocytes
Source: PLoS Genet. 2023 Aug 1;19(8):e1010855. doi: 10.1371/journal.pgen.1010855 (PMC10393158; doi:10.1371/journal.pgen.1010855)
Supplement: S9 Table — (PDF) [file pgen.1010855.s015.pdf]

S9 Table: Oligonucleotide primers used in this study.

Guide RNA	5'-GCTTACCAGTATGACGACGATGG-3'
Single-stranded donor oligonucleotides	5'-CCCTAGAACTGCTTCTTGGAGTGTGCTTACCAGTATGCCGACG CTGGGTACCAGTCCTATTGCACCATCTGCTGTGGGGGGCGTGAA-3'
Dnmt3a^{ADA}_Fwd	5'-GGCATGTGCCAGAACTGTAA-3'
Dnmt3a^{ADA}_Rev	5'-GACTGGTACCCAGCGTCGG-3'
ADD domain cloning_Fwd	5'-CTGGGATCCAGGGAGCGGCTGGTGTATGAG-3'
ADD domain cloning_Rev	5'-CGGGAATTCTTAAAAGAACATCTGGAGTCGAGA-3'
